# Supplementary material for: Structural insights into GrpEL1-mediated nucleotide and substrate release of human mitochondrial Hsp70
Source: Nat Commun. 2024 Dec 30;15:10815. doi: 10.1038/s41467-024-54499-1 (PMC11685456; doi:10.1038/s41467-024-54499-1)
Supplement: Supplementary file 1 — Supplementary Information [file 41467_2024_54499_MOESM1_ESM.pdf]

Supplementary Information

Structural insights into GrpEL1-mediated nucleotide and substrate release of human mitochondrial Hsp70

Authors:

Marc A. Morizono[1], Kelly L. McGuire[1], Natalie I. Birouty[1], and Mark A. Herzik, Jr.[1*]

*Corresponding Author: *mherzik@ucsd.edu*

Affiliations:

[1]Department of Chemistry and Biochemistry, University of California, San Diego, La Jolla, CA, USA

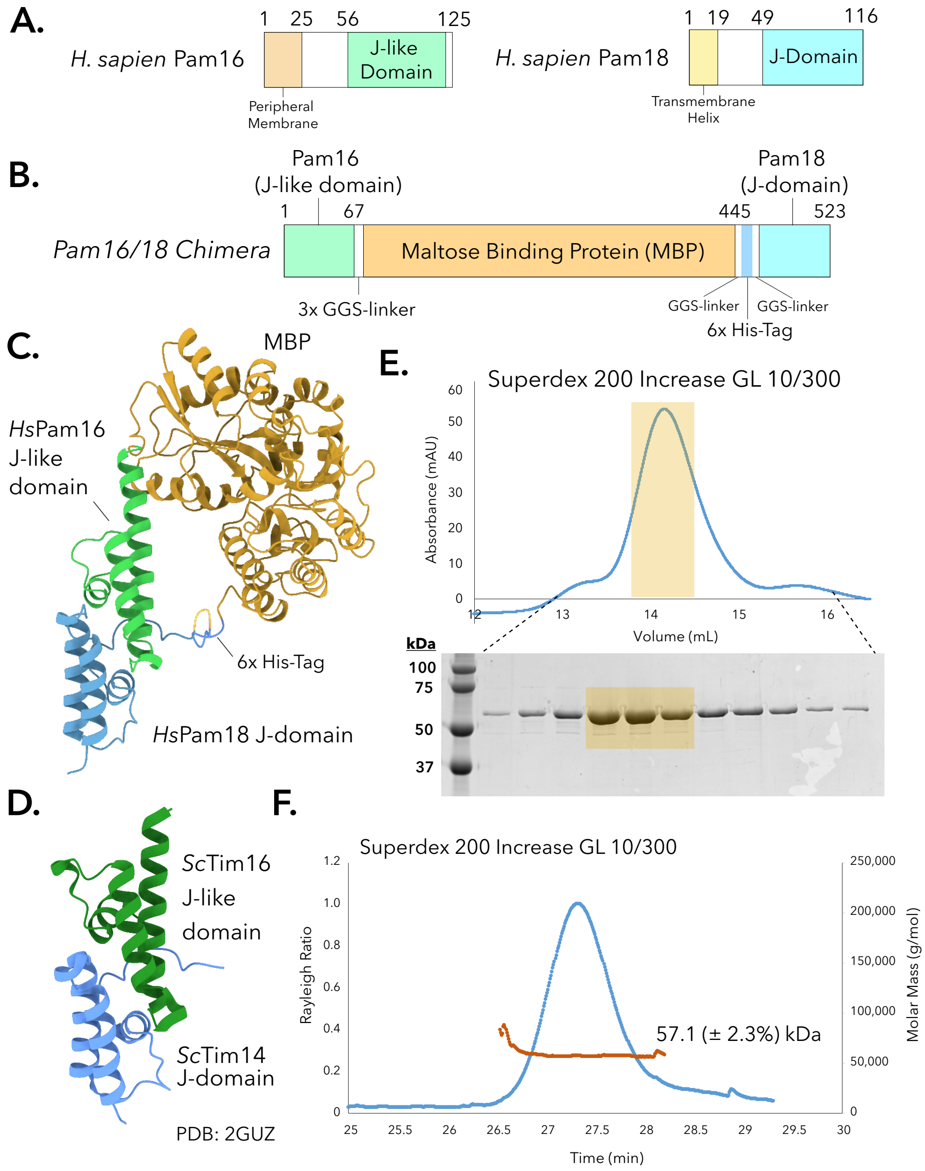

Supplementary Figure 1. Rational construct design and biochemical preparation of a chimeric human Pam16/Pam18 fusion. A. Domain topology of *Hs*Pam16 and *Hs*Pam18. **B.** Domain topology of the designed Pam16/Pam18 chimera fusion. **C.** AlphaFold2 prediction of the Pam16/Pam18 chimera shown in **B**. **D.** Crystal structure of the *Saccharomyces cerevisiae* Tim16 J-like domain (*Hs*Pam16 homolog) and Tim14 J-domain (*Hs*Pam18 homolog) (PDB: 2GUZ). The arrangement of the Tim16/Tim14 heterodimer is similar to the predicted structure of the Pam18/Pam16 chimera. **E.** Preparatory size exclusion chromatogram of the Pam16/Pam18 chimera and SDS-PAGE analysis. The highlighted regions were pooled and concentrated for immediate use or flash frozen using liquid nitrogen. **F.** Size exclusion chromatography multi-angular light scattering (SEC-MALS) analysis of purified Pam16/Pam18 chimera. Expected molecular weight: 57 kDa. The light scattering curve is represented in blue and the molecular weight determination is represented in red (with estimated error shown in parentheses).

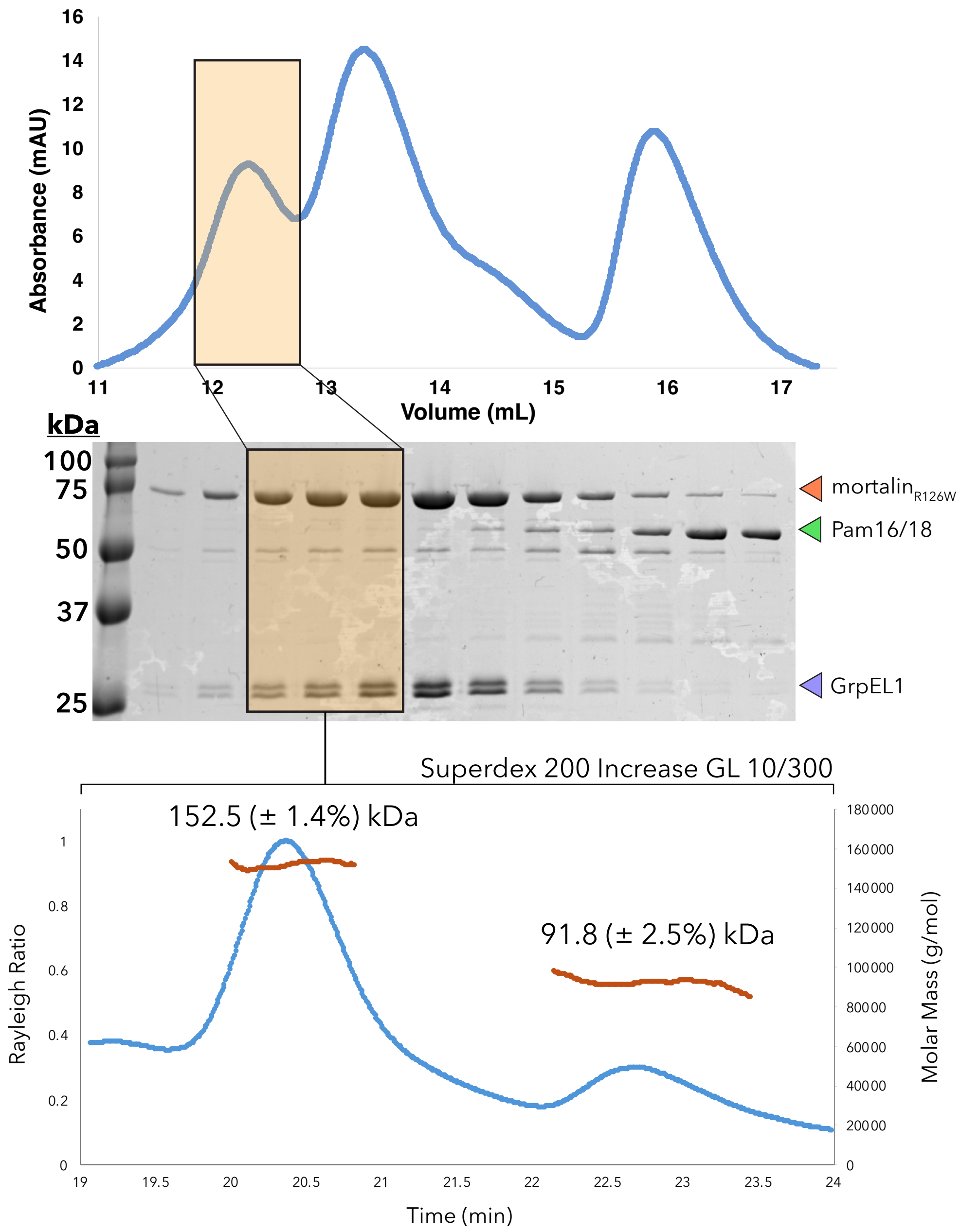

Supplementary Figure 2. Biochemical preparation of the mortalin$_{R126W}$-GrpEL1$_{WT}$ complex. *(top)* Highlighted fractions from a Superdex 200 Increase GL 10/300 SEC run (adapted from Figure 1B) were concentrated to 0.9 mg/mL and subsequently subjected to *(bottom)* SEC-MALS analysis (Superdex 200 Increase GL 10/300 at 0.5 mL/min at room temperature). The higher molecular weight species corresponds to full-length mortalin$_{R126W}$ (70.2 kDa), a GrpEL1 homodimer (47 kDa), and a mortalin$_{R126W}$ IDL-SBD truncation product (28.5 kDa). The lower molecular weight species corresponds to a mortalin$_{R126W}$ NBD (41.8 kDa) and a GrpEL1 dimer (47 kDa). Mortalin: 70.2 kDa, GrpEL1 (monomer): 23.5 kDa, mortalin (NBD): 41.8 kDa, mortalin (IDL-SBD): 28.5 kDa. The light scattering curve is represented in blue and the molecular weight determination is represented in red (with estimated error shown in parentheses).

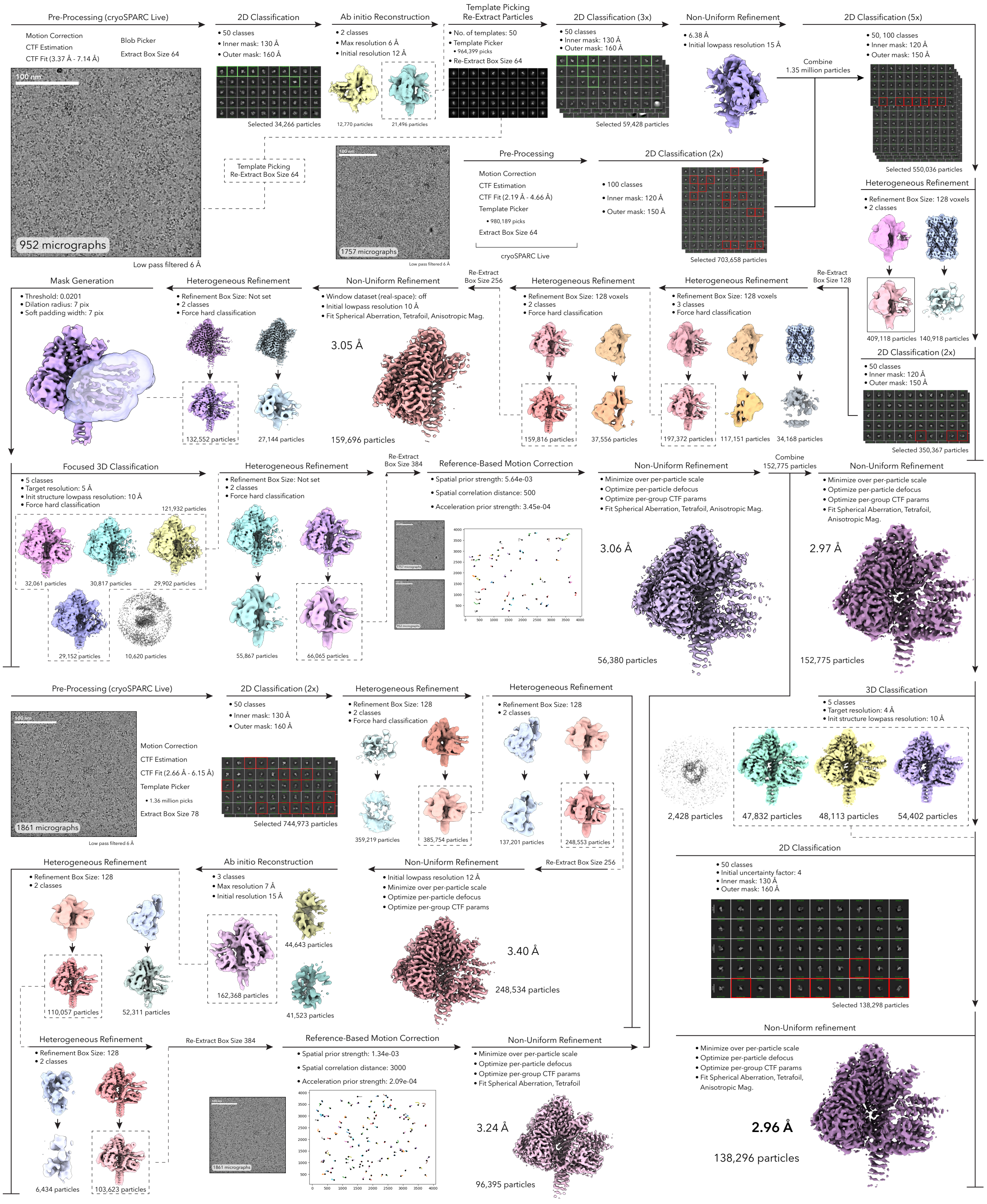

Supplementary Figure 3. CryoEM data processing of the mortalin$_{R126W}$-GrpEL1$_{WT}$ complex. See ***Methods*** for detailed explanation of cryoEM data processing. For 2-D classification, classes highlighted in green were chosen as selected particles; classes highlighted in red were excluded from the selected particles. Relevant parameters for each step are listed accordingly.

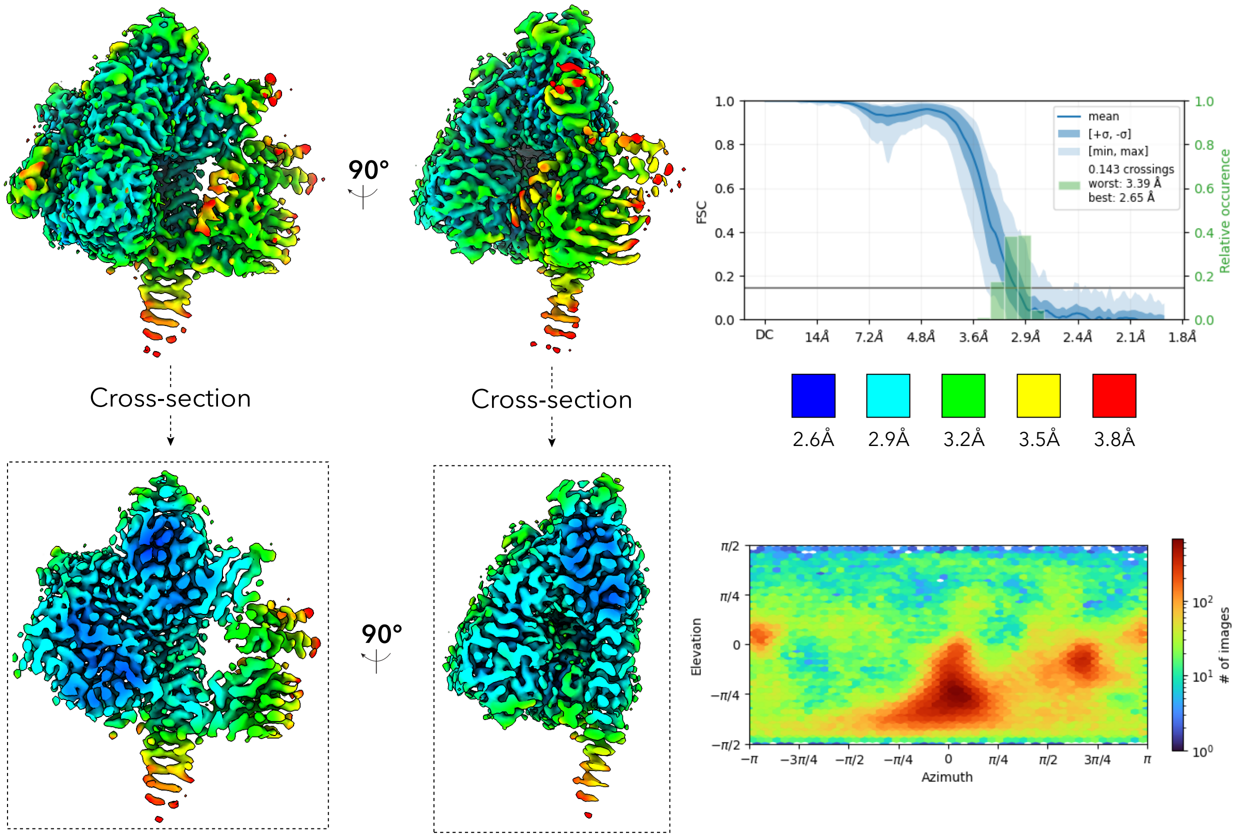

Supplementary Figure 4. Local resolution estimation of the mortalin$_{R126W}$-GrpEL1$_{WT}$ complex. Locally filtered EM density of the mortalin$_{R126W}$-GrpEL1$_{WT}$ complex colored by local resolution. Cross-sections of the EM density are shown below each view. 3-D Fourier shell correlation (FSC) plots generated from the independent half maps contributing to the ~2.96 Å mortalin-GrpEL1$_{WT}$ map. An elevation plot describing the angular distribution of particles used in the final reconstruction is shown.

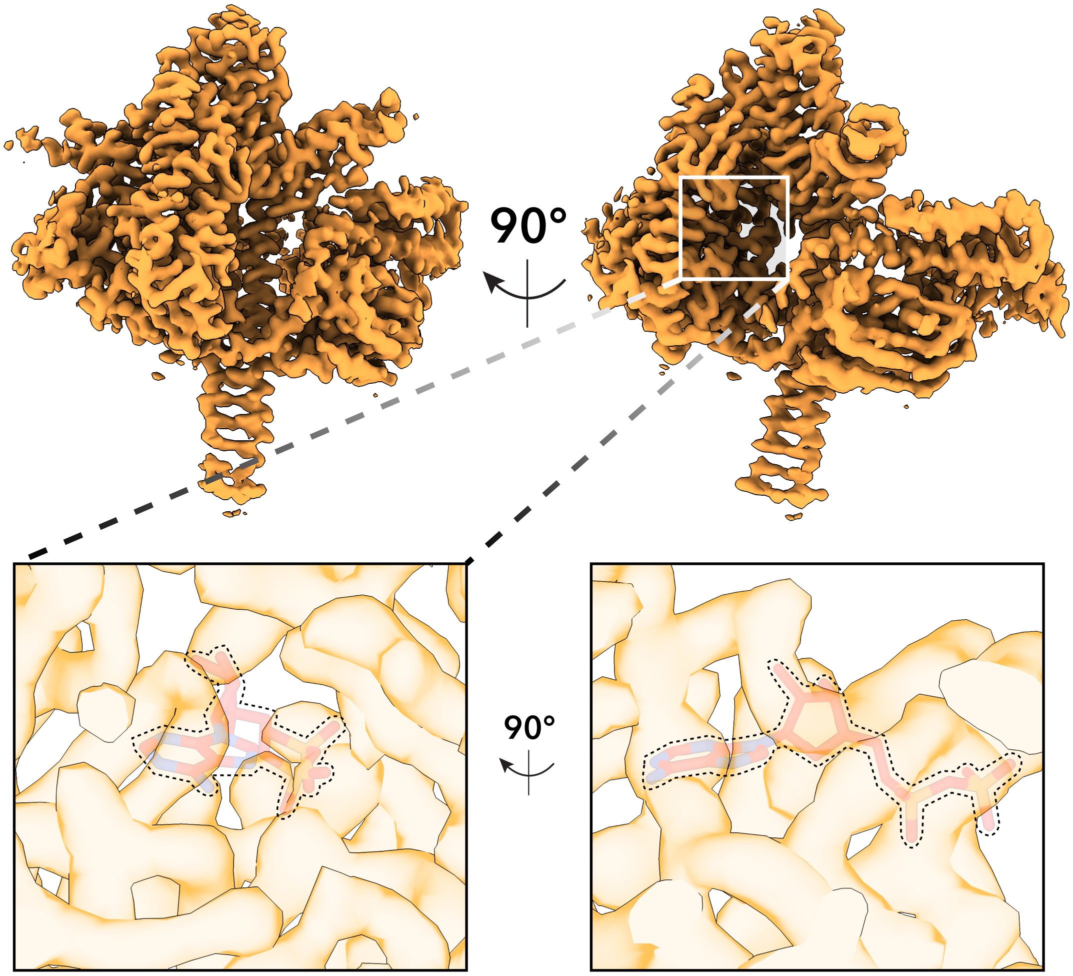

Supplementary Figure 5. Visualization of the nucleotide binding pocket in the mortalin$_{R126W}$-GrpEL1$_{WT}$ complex. DeepEMhancer-sharpened map of the *Hs*mortalin$_{R126W}$-GrpEL1 complex. Density for the putative ADP binding site is shown as a transparent, outlined model (PDB: 5OBW). No electron density for nucleotide is observed in the *Hs*mortalin$_{R126W}$-GrpEL1 complex.

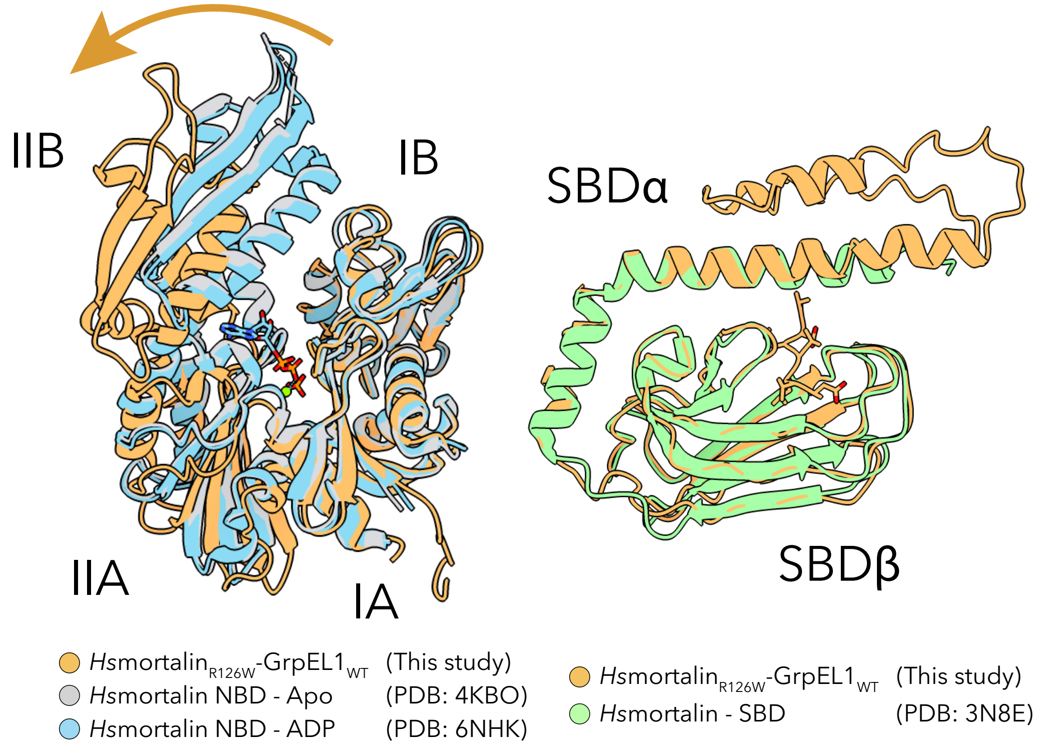

Supplementary Figure 6. Comparison of the NBD and SBD domains in the mortalin$_{R126W}$-GrpEL1$_{WT}$ complex with mortalin crystal structures. *(left)* Superposition of the NBD from *Hs*mortalin$_{R126W}$ complexed with GrpEL1$_{WT}$ (this study) and *Hs*mortalin NBD crystal structures in either apo (PDB: 4KBO) or ADP-bound (PDB: 6NHK) states. *(right)* Superposition of the substrate-bound SBD from *Hs*mortalin$_{R126W}$ complexed with GrpEL1$_{WT}$ (this study) and the *Hs*mortalin SBD crystal structure (PDB: 3N8E).

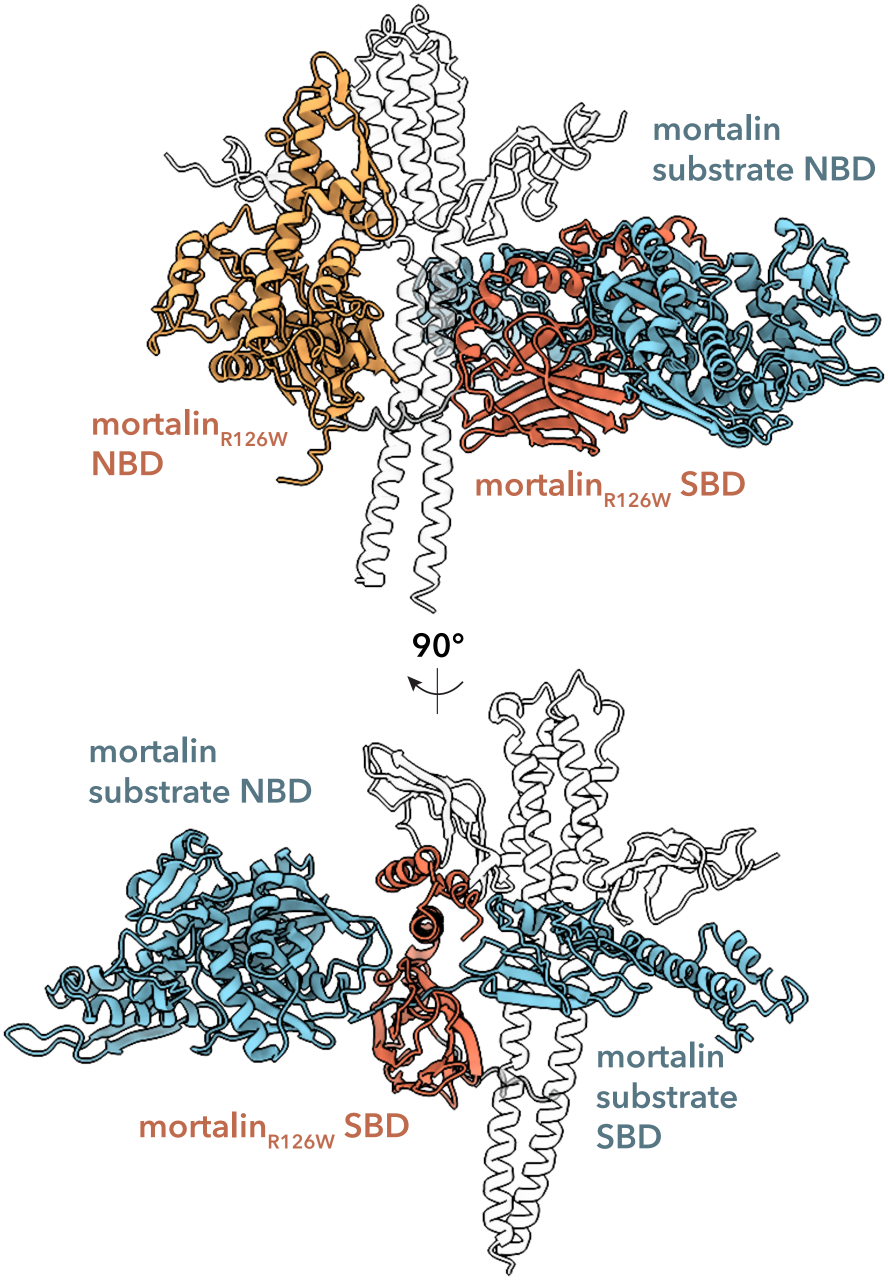

Supplementary Figure 7. Mortalin$_{R126W}$ in complex with GrpEL1 can accommodate full-length mortalin as a substrate. The mortalin$_{R126W}$ SBD was rigid-body docked into the posterior substrate EM density with a low-pass filtered mortalin$_{R126W}$-GrpEL1$_{WT}$ map, as shown in **Figure 4B**. The NBD from the mortalin$_{R126W}$-GrpEL1$_{WT}$ structure was separated and aligned with the IDL in the mortalin$_{R126W}$-GrpEL1$_{WT}$ complex structure. The NBD was oriented to mimic substrate binding of a full-length mortalin unit within the mortalin SBD of the mortalin$_{R126W}$-GrpEL1$_{WT}$ complex structure. An orthogonal view shows that the mortalin SBD could accommodate full-length mortalin.

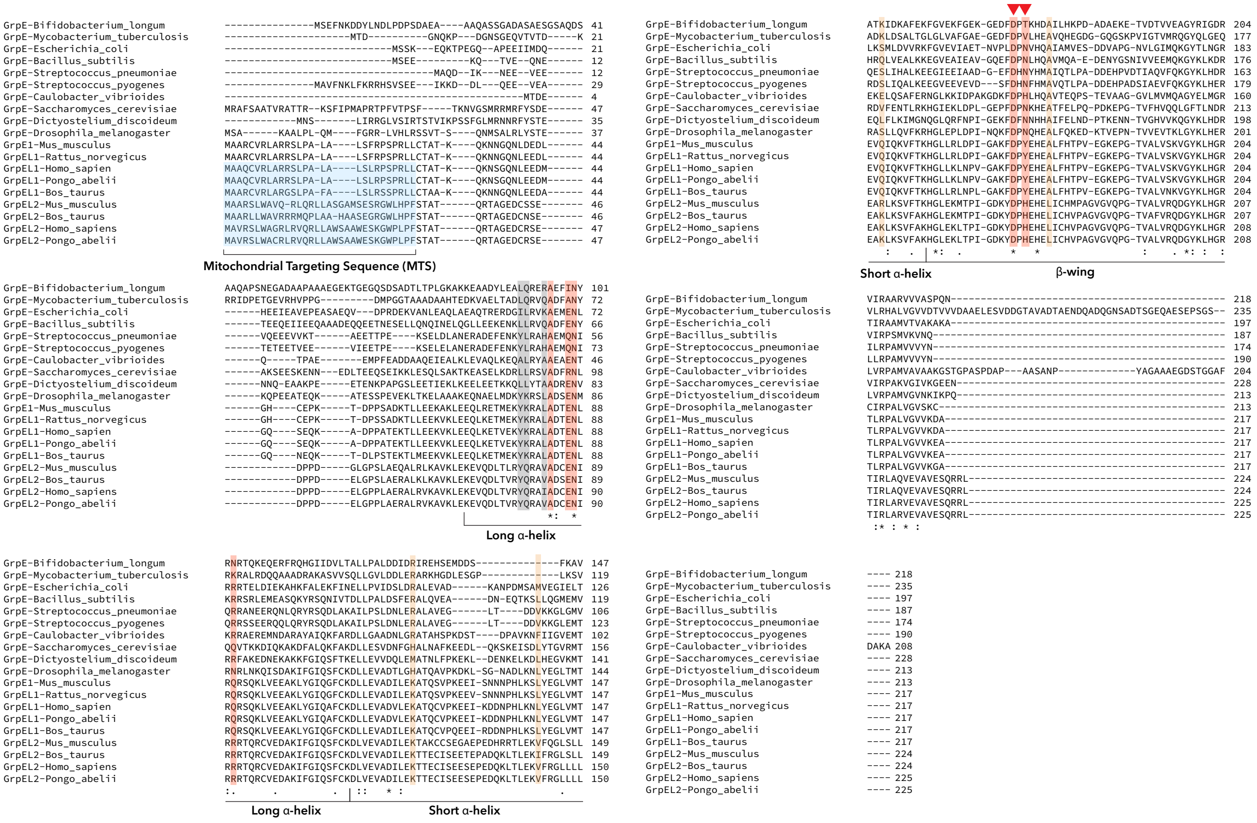

Supplementary Figure 8. Multiple sequence alignment of GrpE-like homologs. Multiple sequence alignment of 19 GrpE-like homologs. Residues that interact with the mortalin NBD are colored in light orange, residues that interact with the mortalin IDL are colored in grey, residues that interact with the SBD are colored in red. D171 and Y173 that interact with the mortalin SBDα lid are indicated with a red triangle. The mitochondrial targeting sequence (MTS) is colored in light blue in mitochondrially targeted GrpE-like species. Residues that are fully conserved across species are noted with an asterisk (*). Residues with strong conservation of similar properties are noted with a colon (:). Residues with weak conservation of similar properties are noted with a period (.). Multiple sequence aligned was performed in Clustal Omega. *Bifidobacterium longum* (entry: Q8G6W2), *Mycobacterium tuberculosis* (entry: P9WMT5), *Escherichia coli* (entry: P09372), *Bacillus subtilis* (entry: P15874), *Streptococcus pneumoniae* (entry: Q97S73), *Streptococcus pyogenes* (entry: Q5XAD5), *Caulobacter vibrio* (entry: B8GXP4), *Saccharomyces cerevisiae* (entry: P38523), *Dictyostelium discoideum* (entry: Q54QF9), *Drosophila melanogaster* (entry: P48604), *Mus musculus* (GrpEL1) (entry: Q99LP6), *Rattus norvegicus* (entry: P97576), *Homo sapien* (GrpEL1) (entry: Q9HAV7), *Pongo abelii* (GrpEL1) (entry: Q5RA81), *Bos taurus* (GrpEL1) (entry: Q3SZC1), *Mus musculus* (GrpEL2) (entry: O88396), *Bos taurus* (GrpEL2) (entry: Q0P5N5), *Homo sapien* (GrpEL2) (entry: Q8TAA5), *Pongo abelii* (GrpEL2) (entry: Q5R435).

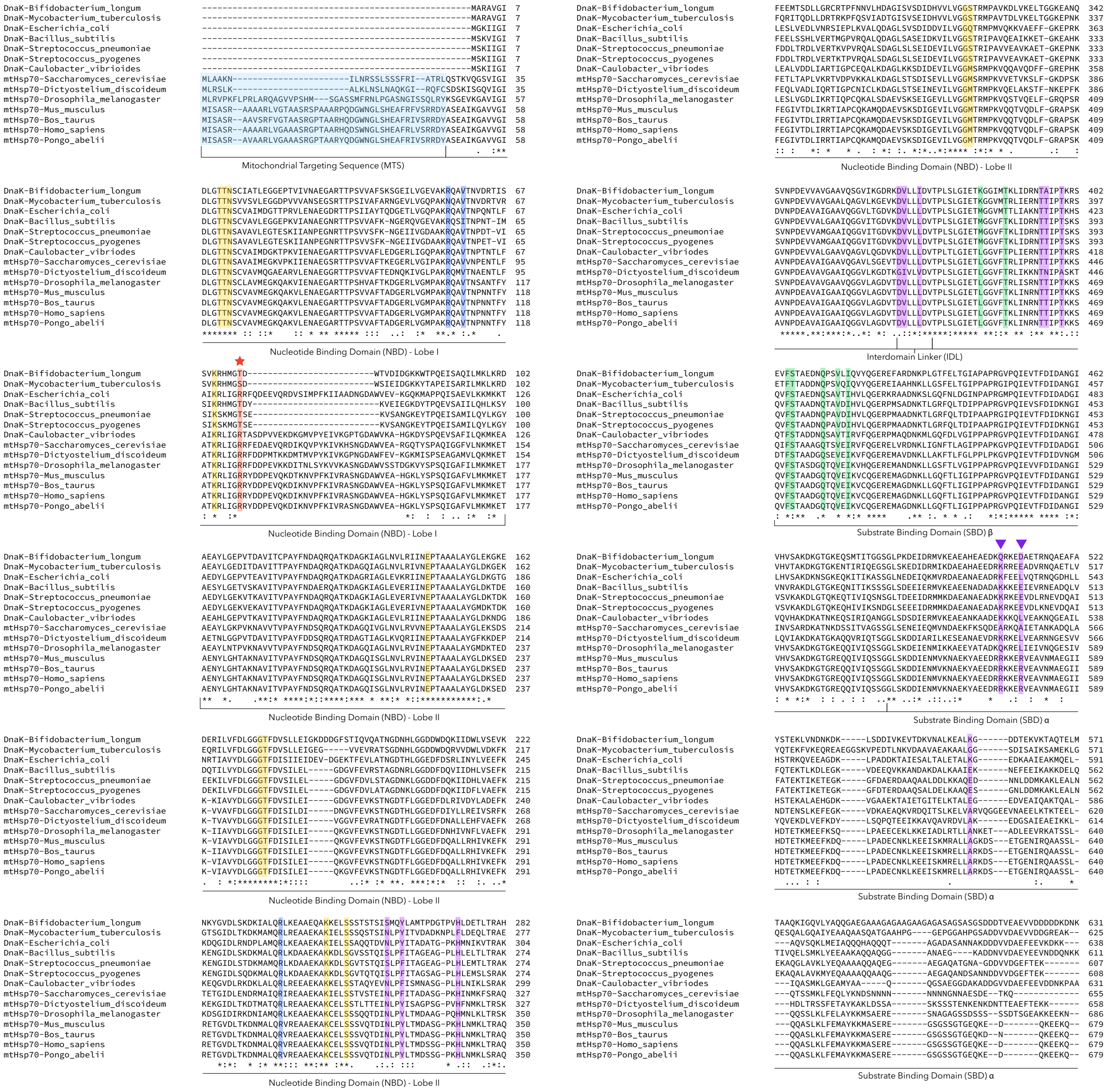

Supplementary Figure 9. Multiple sequence alignment of mitochondrial (mt)Hsp70 homologs. Multiple sequence alignment of 14 mtHsp70 homologs. Residues that interact with GrpEL1-A are colored in dark blue and residues that interact with GrpEL1-B are colored in purple. R574 and R578 that interact with GrpEL1-B are indicated with a purple triangle. R126, mutated to R126W in this study, is highlighted in orange and indicated with an orange star. Residues that interact with bound substrate are colored in green. Residues in the nucleotide binding site are colored in gold. The mitochondrial targeting sequence (MTS) is colored in light blue in mitochondrially targeted mtHsp70 species. Residues that are fully conserved across species are noted with an asterisk (*). Residues with strong conservation of similar properties are noted with a colon (:). Residues with weak conservation of similar properties are noted with a period (.). Multiple sequence alignment was performed in Clustal Omega. *Bifidobacterium longum* (entry: B7GT47), *Mycobacterium tuberculosis* (entry: P9WMJ9), *Escherichia coli* (entry: P0A6Y8), *Bacillus subtilis* (entry: P17820), *Streptococcus pneumoniae* (entry: Q8CWT3), *Streptococcus pyogenes* (entry: P0C0C6), *Caulobacter vibrioides* (entry: P20442), *Saccharomyces cerevisiae*

(entry: P0CS90), *Dictyostelium discoideum* (entry: Q8I0H7), *Drosophila melanogaster* (entry: P82910), *Mus musculus* (entry: P38647), *Bos taurus* (entry: Q3ZCH0), *Homo sapien* (entry: P38646), *Pongo abelii* (entry: Q5R511).

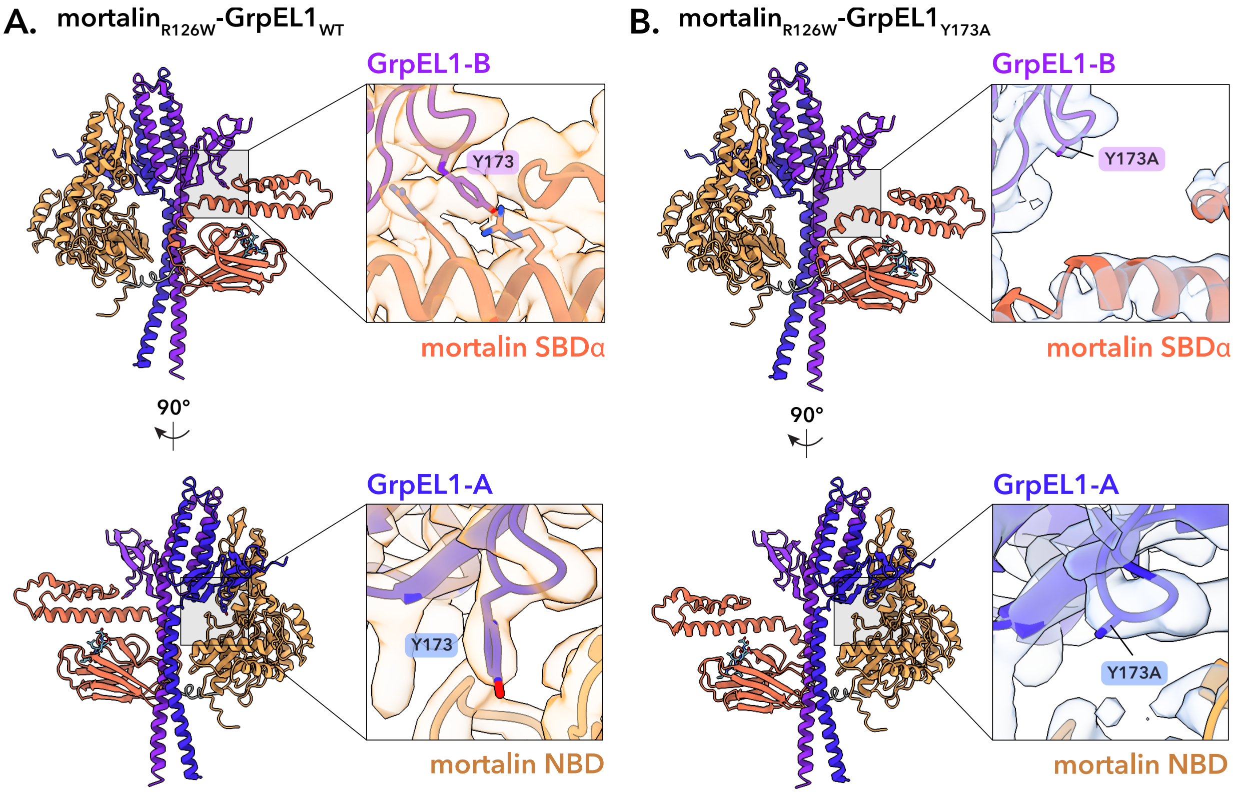

Supplementary Figure 10. GrpEL1 has interaction interfaces that uniquely interact with either the mortalin NBD or SBD. A. Visualization of Y173 in both GrpEL1-B *(top)* and GrpEL1-A *(bottom)* in the mortalin$_{R126W}$-GrpEL1$_{WT}$ structure. While Y173 makes close contacts with mortalin in GrpEL1-B, Y173 does not appear to contact mortalin$_{R126W}$ in GrpEL1-A. The mortalin$_{R126W}$-GrpEL1 map was modified using DeepEMhancer for visualization. **B)** Visualization of Y173A in both GrpEL1-B *(top)* and GrpEL1-A *(bottom)* in the mortalin$_{R126W}$-GrpEL1$_{Y173A}$ structure. Large separation is observed between Y173A in GrpEL1-B and mortalin$_{R126W}$. Y173A in GrpEL1-A does not appear to contact mortalin. The mortalin$_{R126W}$-GrpEL1$_{Y173A}$ map was modified using DeepEMhancer for visualization.

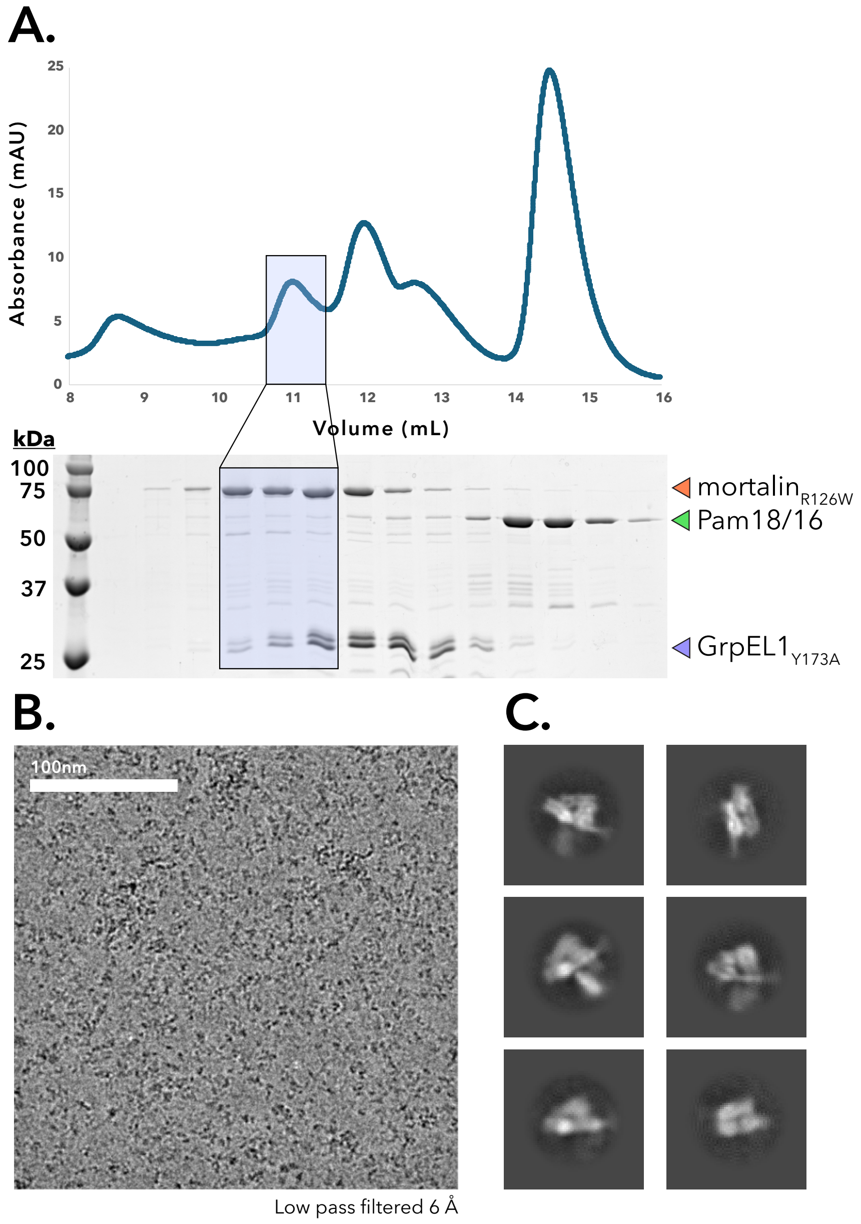

Supplementary Figure 11. Biochemical preparation of the mortalin$_{R126W}$-GrpEL1$_{Y173A}$ complex. A. Size exclusion chromatogram (A_{280}) and corresponding SDS-PAGE analysis. The left-most peak was concentrated and vitrified onto cryoEM grids. **B.** Representative micrograph of the mortalin$_{R126W}$-GrpEL1$_{Y173A}$ complex. Micrograph was low-pass filtered to 6 Å for clarity. **C.** Representative two-dimensional (2-D) classes of the mortalin$_{R126W}$-GrpEL1$_{Y173A}$ complex.

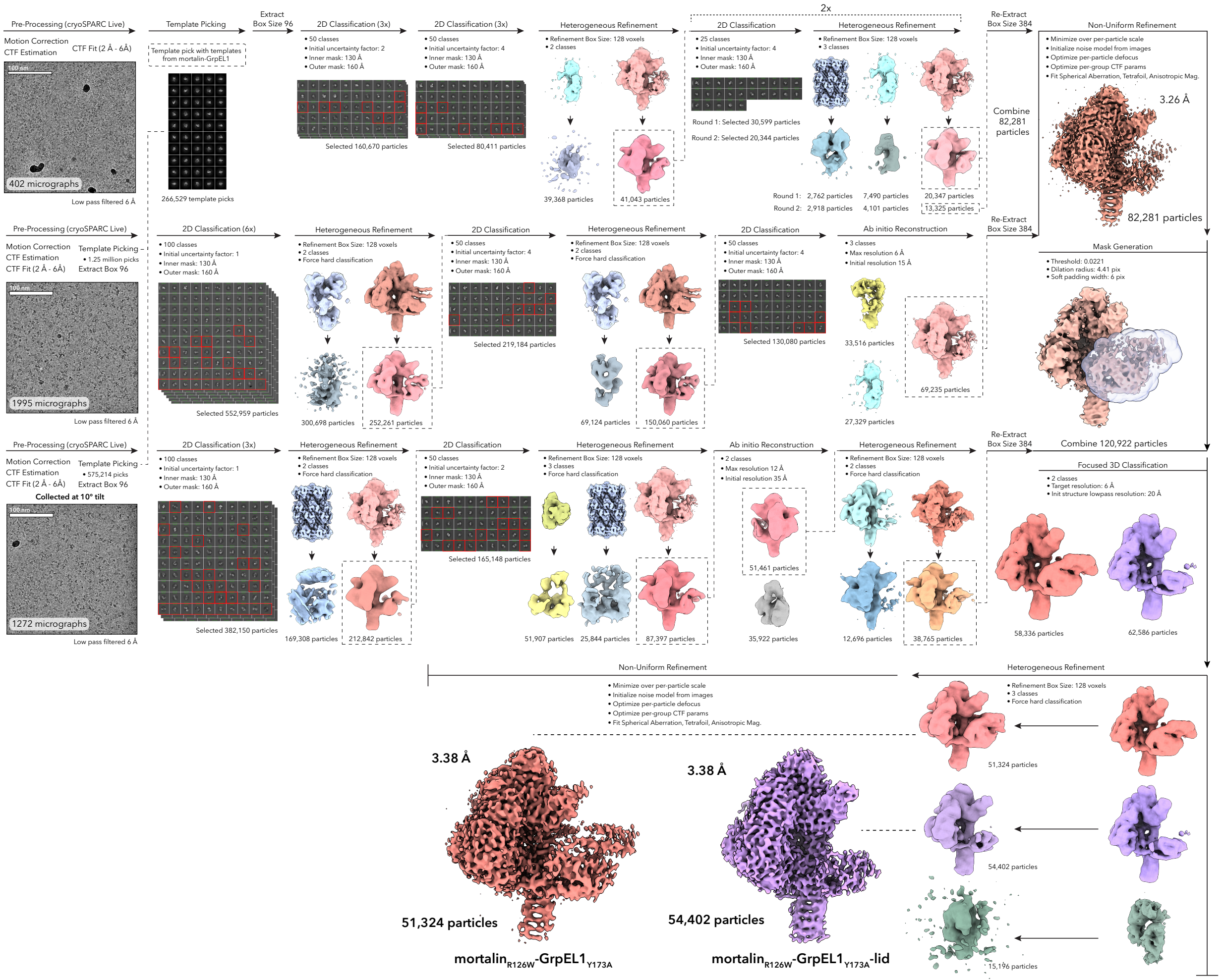

Supplementary Figure 12. CryoEM data processing of the mortalin$_{R126W}$-GrpEL1$_{Y173A}$ complexes. See ***Methods*** for detailed explanation of cryoEM data processing. For 2-D classification classes highlighted in red were excluded from the selected particles. Relevant parameters for each step are listed accordingly.

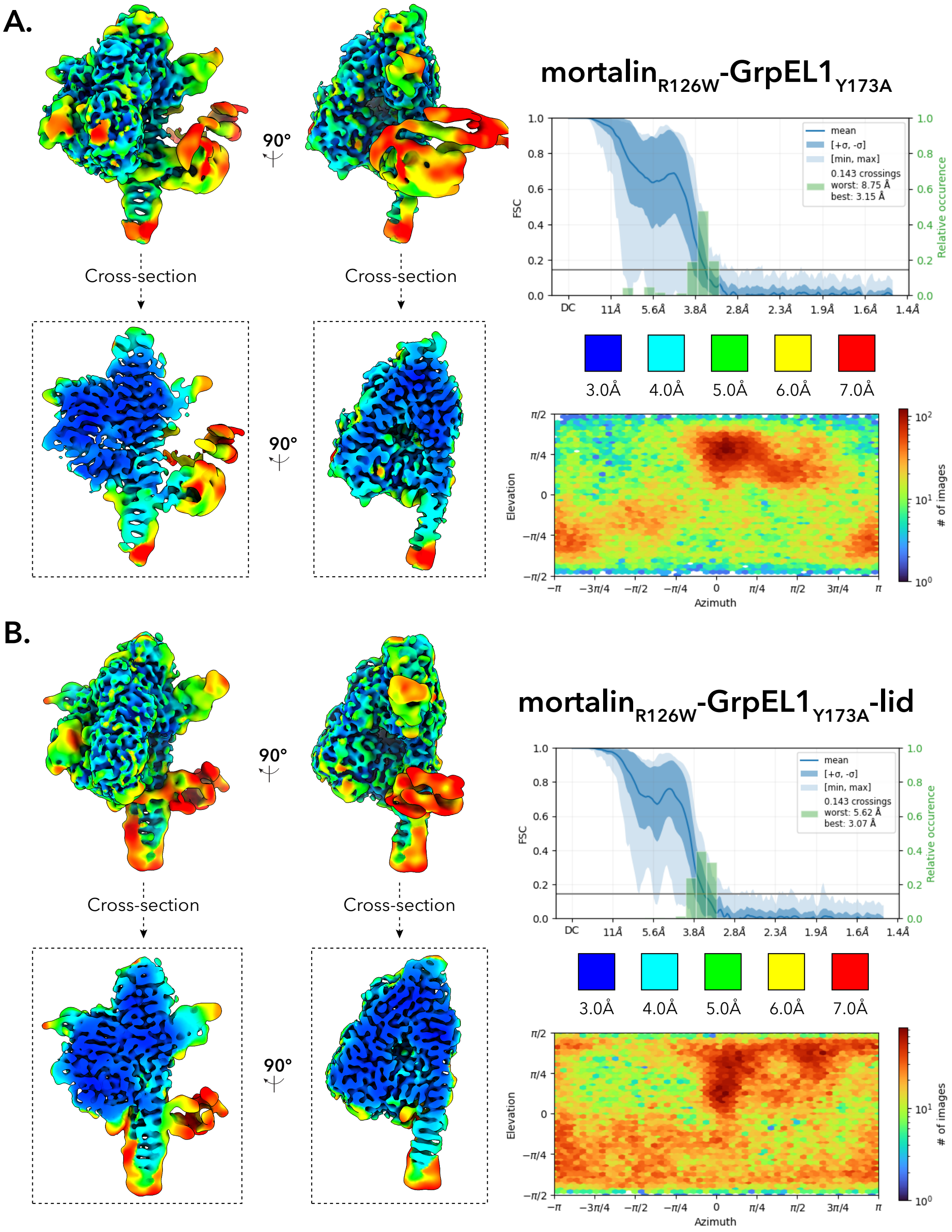

Supplementary Figure 13. Local resolution estimation of the mortalin$_{R126W}$-GrpEL1$_{Y173A}$ and mortalin$_{R126W}$-GrpEL1$_{Y173A}$–lid complexes. A. Locally filtered EM density of the mortalin$_{R126W}$-GrpEL1$_{Y173A}$ complex colored by local resolution. Cross-sections of the EM density are shown below each view. 3-D Fourier shell correlation (FSC) plots were generated from the independent half maps contributing to the ~3.38 Å mortalin-GrpEL1$_{Y173A}$ map. An elevation plot describing the angular distribution of particles used in the final reconstruction is shown. **B.** Locally filtered EM density of the mortalin$_{R126W}$-GrpEL1$_{Y173A}$-lid complex colored by local resolution. Cross-sections of the EM density are shown below each view. 3-D Fourier shell correlation (FSC) plots were generated from the independent half maps contributing to the ~3.38 Å mortalin$_{R126W}$-GrpEL1$_{Y173A}$-

lid map. An elevation plot describing the angular distribution of particles used in the final reconstruction is shown.

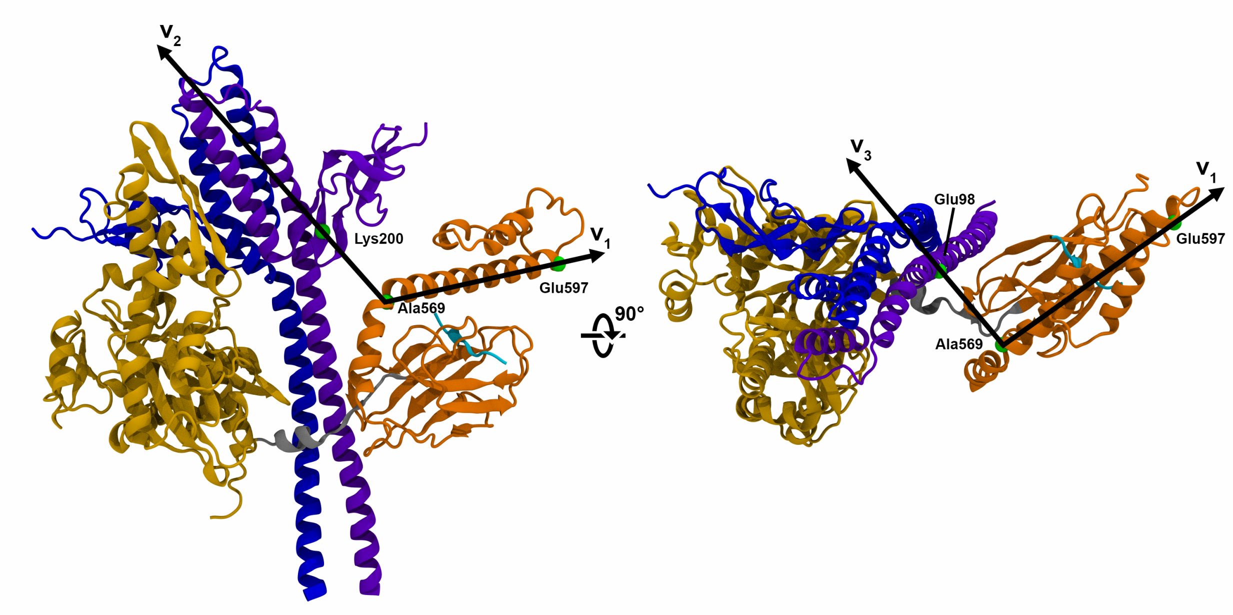

Supplementary Figure 14. Defining vectors for medial and lateral motions.[60] Vectors were defined to measure the changes in medial and lateral motions throughout the all-atom simulations of mortalin$_{R126W}$-GrpEL1$_{WT}$ and mortalin$_{R126W}$-GrpEL1$_{Y173A}$. *(left)* Vector v_1, defined by the Cα's of Ala569 and Glu597 in the mortalin SBD, and vector v_2, defined by the Cα's of Ala569 in the mortalin SBD and Lys200 in GrpEL1-B, were used to measure medial motions in all-atom simulations. *(right)* Vector v_1 and vector v_3, defined by the Cα's of Ala569 and Glu98 in GrpEL1-B, were used to measure lateral motions in all-atom simulations.

.

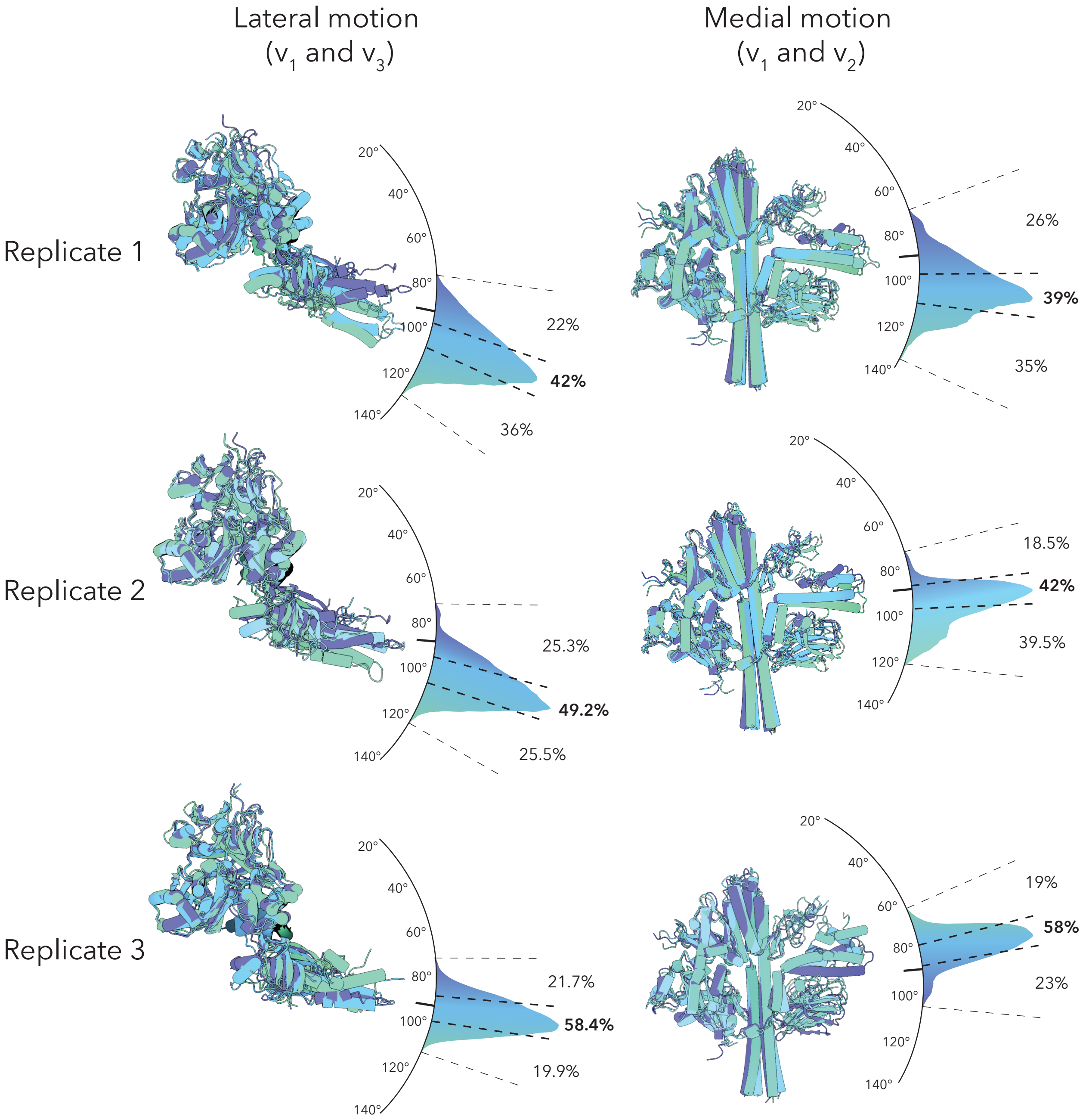

Supplementary Figure 15. Angular analysis of lateral and medial motions in mortalin$_{R126W}$-GrpEL1$_{WT}$ all-atom simulations. Changes in lateral and medial motions, defined by vectors v_1 and v_3, and v_1 and v_2 respectively **(Supplementary Figure 14)**, were monitored throughout three, 150ns replicates of all-atom simulations for mortalin$_{R126W}$-GrpEL1$_{WT}$. Representative snapshots of substates, in dark blue, light blue, and seafoam green, correspond to the colored regions on the angular plot and are representative of the substate in the regions between the dashed lines. Percentage of trajectory in each region are annotated on the angular plot. The angle of the initial substate is denoted by a black line on the angular plot.

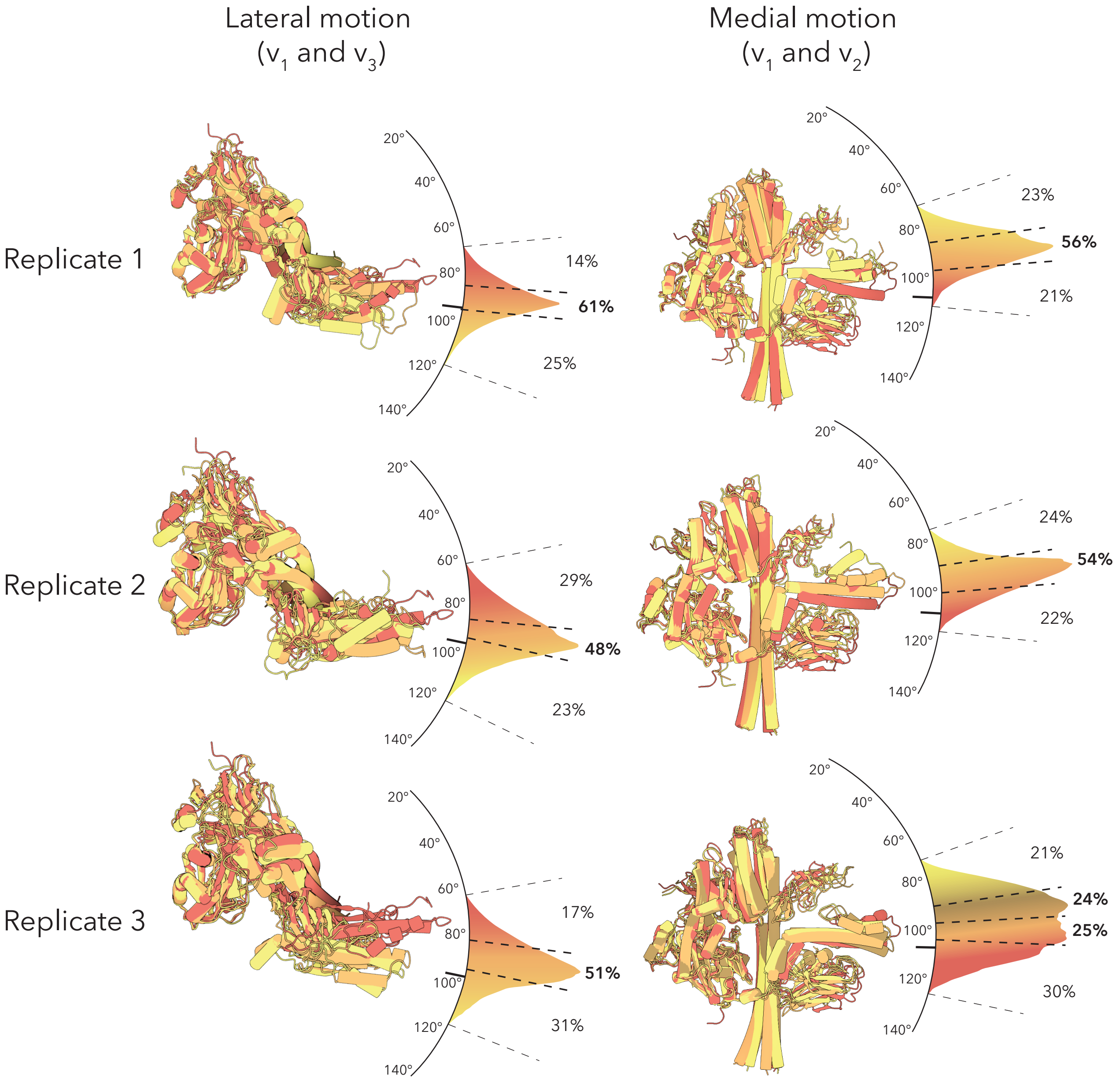

Supplementary Figure 16. Angular analysis of lateral and medial motions in mortalin$_{R126W}$-GrpEL1$_{Y173A}$ all-atom simulations. Changes in lateral and medial motions, defined by vectors v_1 and v_3, and v_1 and v_2 respectively **(Supplementary Figure 14)**, were monitored throughout three, 150ns replicates of all-atom simulations for mortalin$_{R126W}$-GrpEL1$_{Y173A}$. Representative snapshots of substates, in yellow, orange, red, and brown correspond to the colored regions on the angular plot and are representative of the substate in the regions between the dashed lines. Percentage of trajectory in each region are annotated on the angular plot. The angle of the initial substate is denoted by a black line on the angular plot.

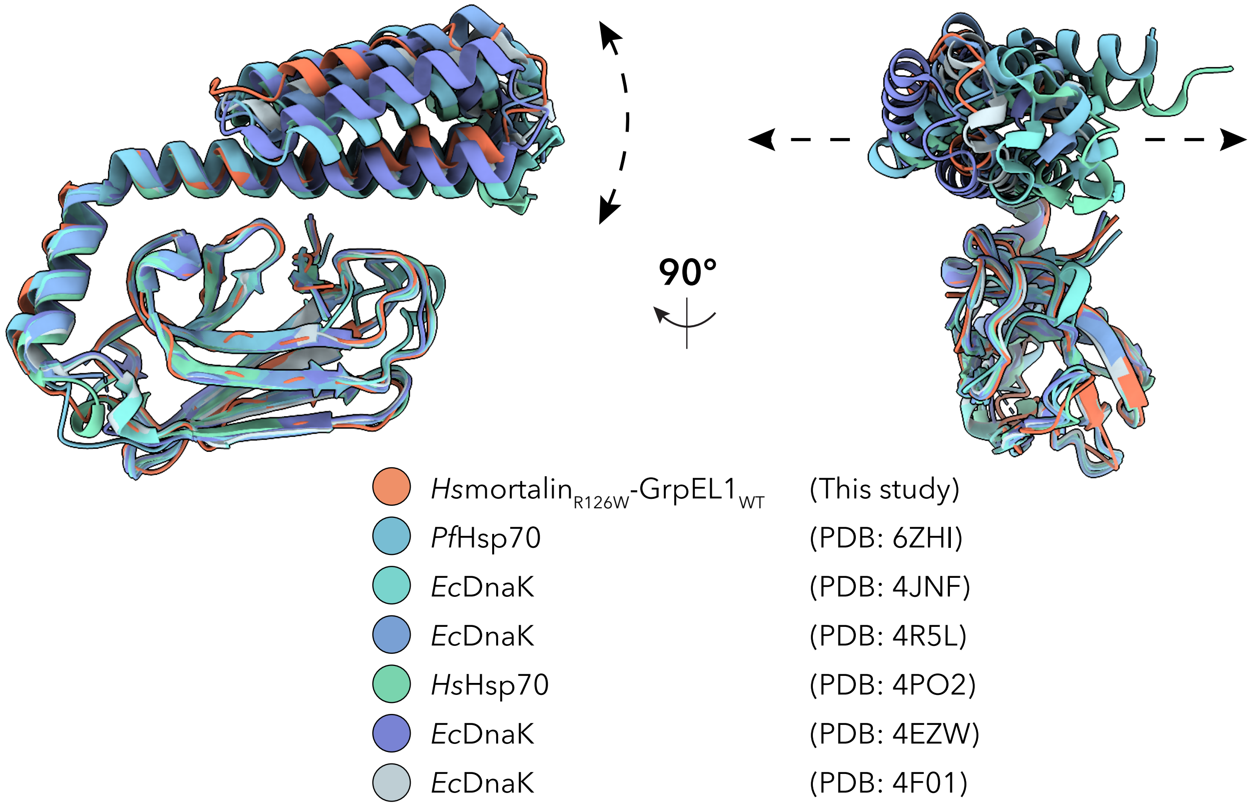

Supplementary Figure 17. Comparison of SBDα in mortalin$_{R126W}$-GrpEL1$_{WT}$ with existing Hsp70 crystal structures. The SBDβ subdomain (residues 440-551) in mortalin$_{R126W}$-GrpEL1$_{WT}$ were aligned to the SBDβ subdomains of available Hsp70 structures (PDB IDs are indicated in the figure) to exemplify the flexibility in the SBDα across these structures.

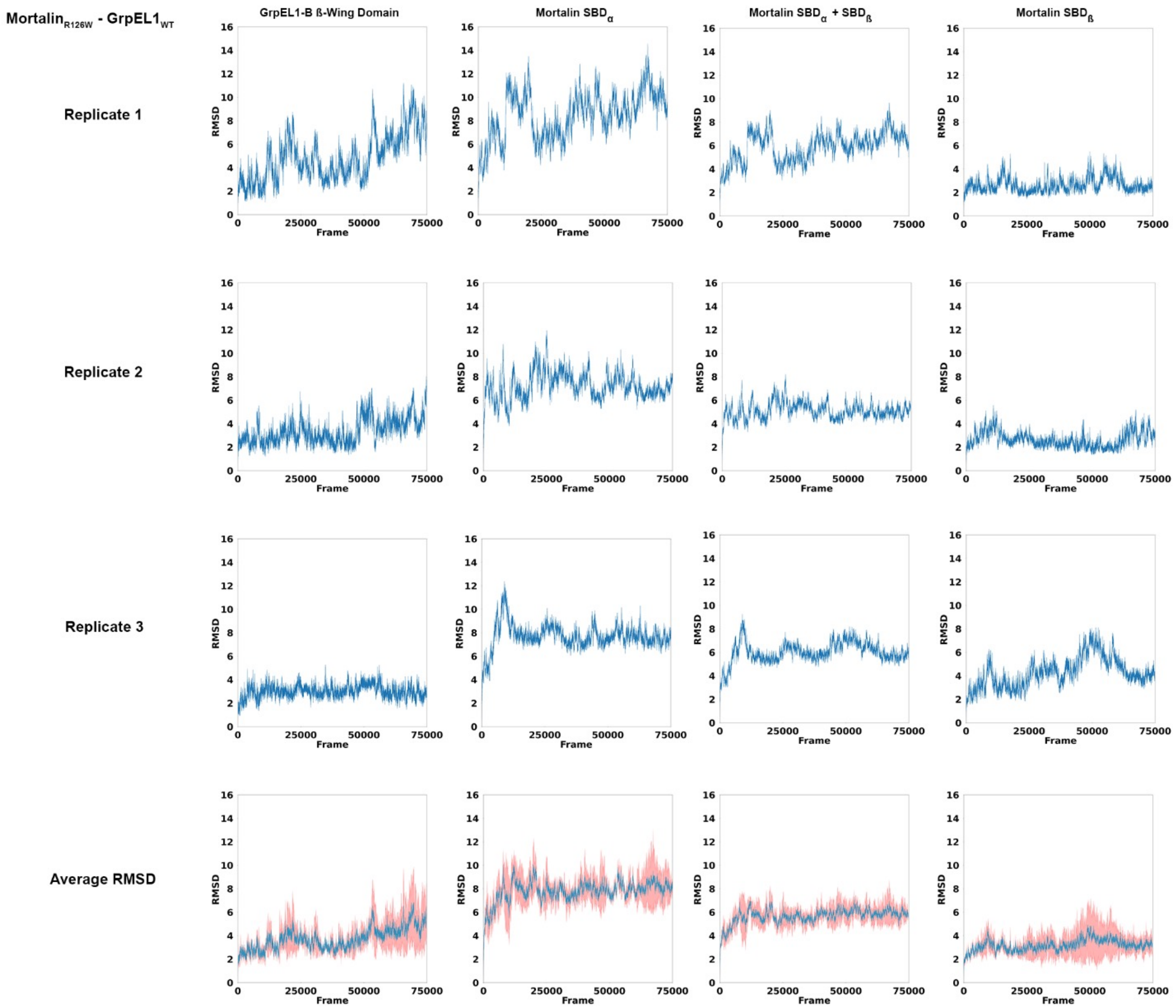

Supplementary Figure 18. RMSD analysis of individual domains in mortalin$_{R126W}$-GrpEL1$_{WT}$. RMSD analyses were carried out on the GrpEL1-B β-wing, the SBDα lid, the SBDβ domain, and the SBDα lid + SBDβ domains throughout the all-atom molecular dynamics simulations. Average RMSDs with standard deviations (shown in red) are included. Domain boundaries used for analysis are described in Methods.

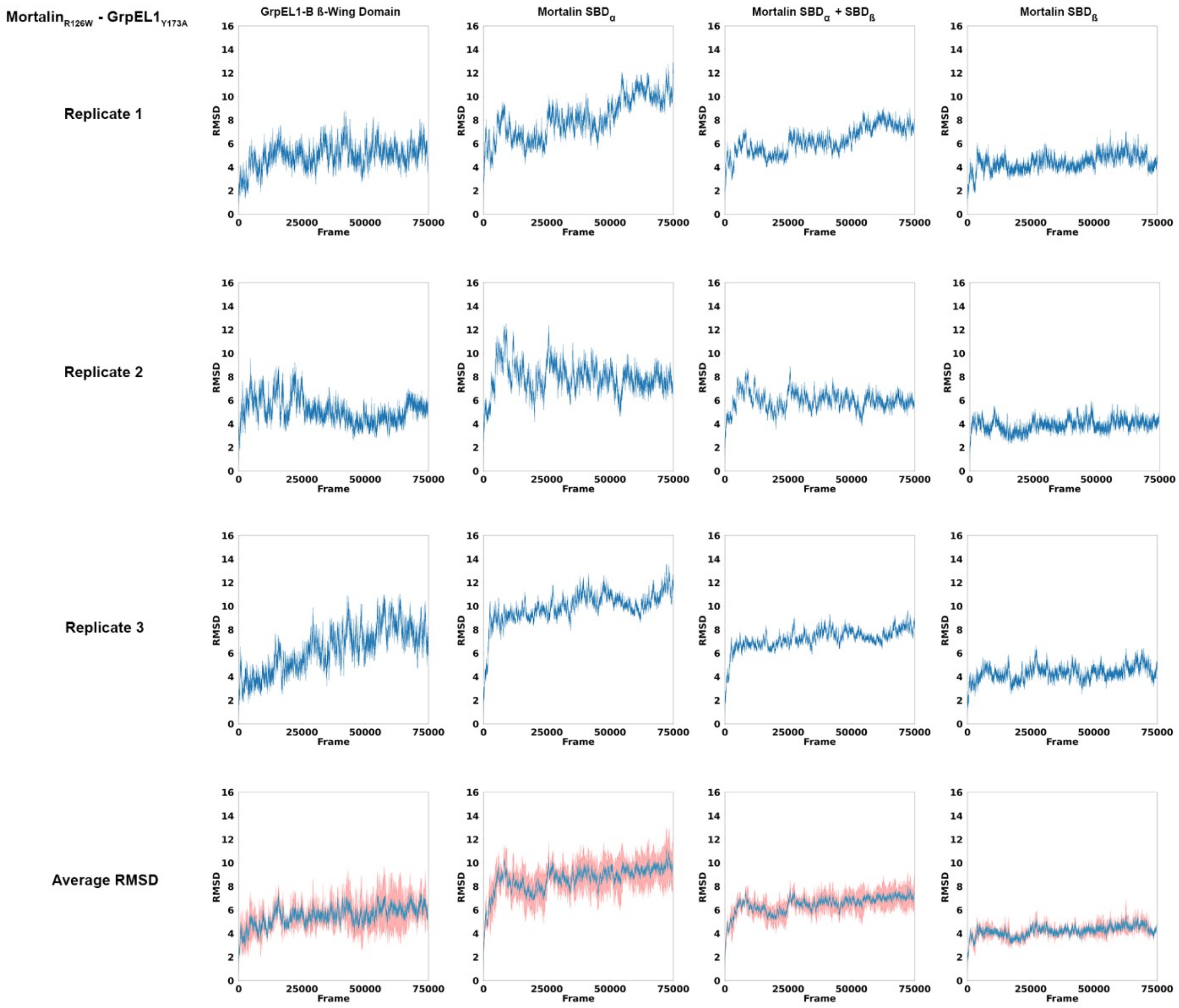

Supplementary Figure 19. RMSD analysis of individual domains in mortalin$_{R126W}$-GrpEL1$_{Y173A}$. RMSD analyses were carried out on the GrpEL1-B β-wing, the SBDα lid, the SBDβ domain, and the SBDα lid + SBDβ domains throughout the all-atom molecular dynamics simulations. Average RMSDs with standard deviations (shown in red) are included. Domain boundaries used for analysis are described in Methods.

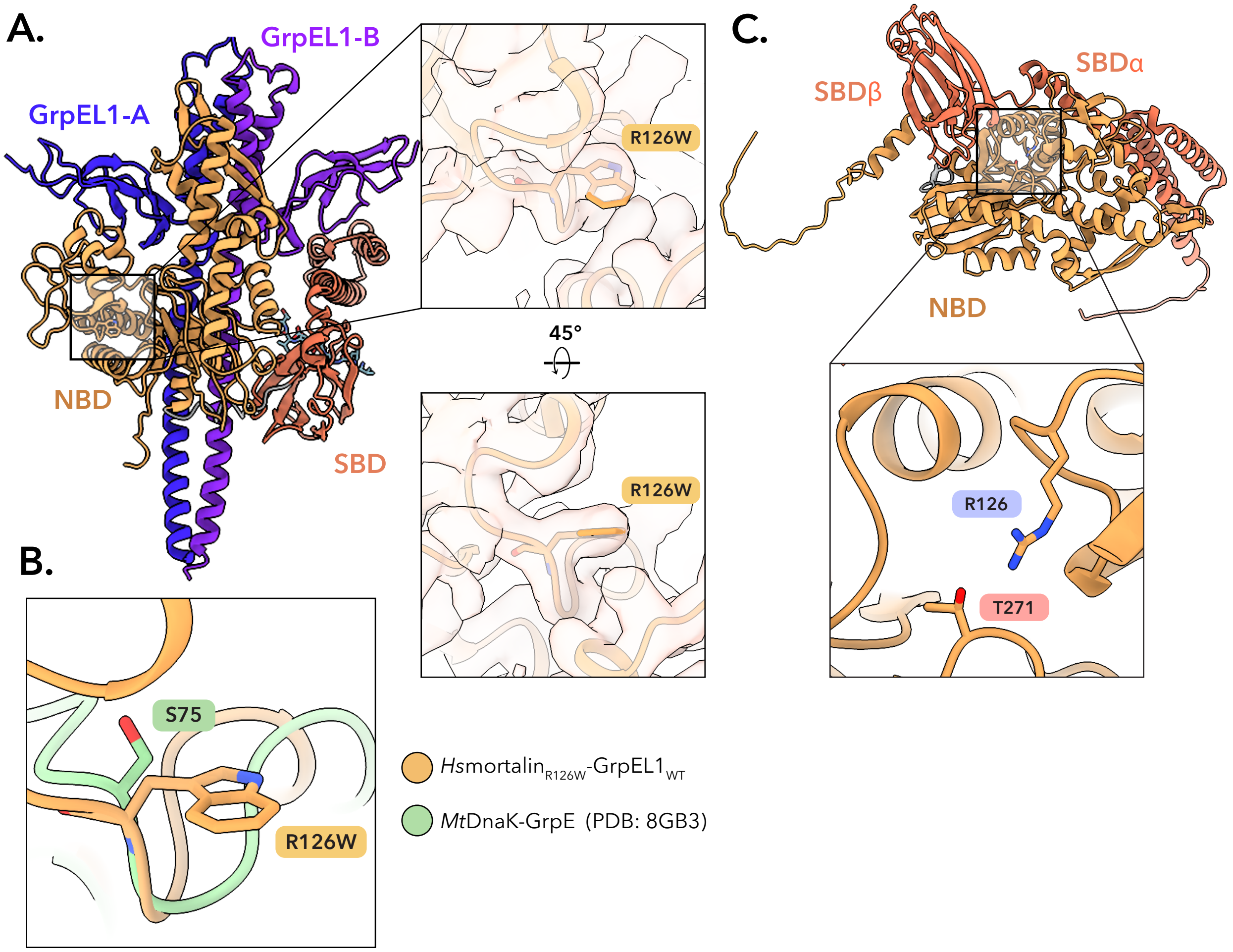

Supplementary Figure 20. Structural analysis of R126W in the mortalin$_{R126W}$-GrpEL1$_{WT}$ complex cryoEM structure. A. R126W does not appear to make additional contacts within the NBD of mortalin$_{R126W}$ when complexed with GrpEL1. **B.** Superposition of *Hs*mortalin$_{R126W}$-GrpEL1$_{WT}$ with *Mt*DnaK-GrpE (PDB: 8GB3). S75 in *Mt*DnaK, corresponding to R126 in *Hs*mortalin, does not appear to contribute to NBD stabilization or complex formation in *Mt*DnaK-GrpE. **C.** AlphaFold2 predicted structure of *Hs*mortalin. In the apo-nucleotide state, R126 may interact with T271 to stabilize interactions across the IB and IIB NBD lobes that would be absent in the R126W mutant.

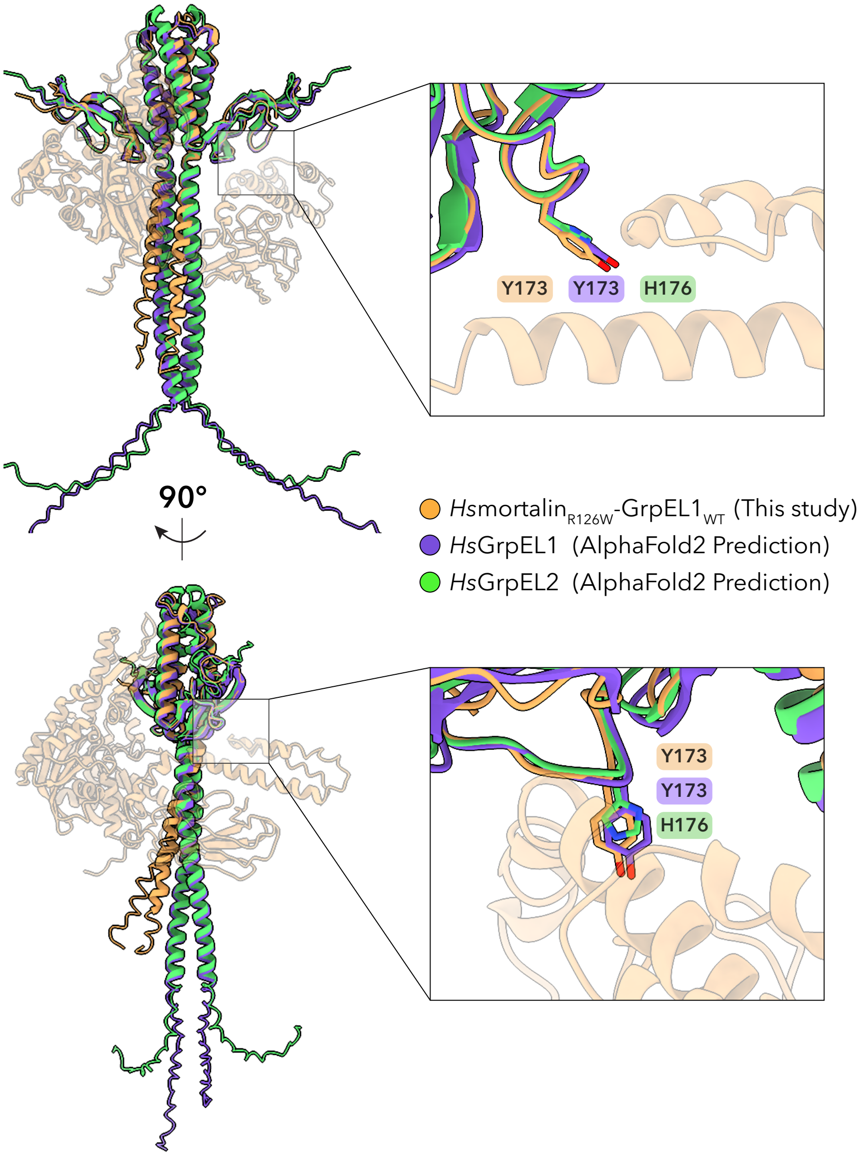

Supplementary Figure 21. Structural comparisons between mortalin$_{R126W}$-GrpEL1$_{WT}$, *Hs*GrpEL1, and *Hs*GrpEL2. GrpEL1-A and GrpEL1-B in the mortalin$_{R126W}$-GrpEL1$_{WT}$ structure were aligned to the AlphaFold2[47] predicted structures of *Hs*GrpEL1 and *Hs*GrpEL2. While Y173 is substituted for H176 in GrpEL2, the positioning of H176 is analogous to Y173 in the mortalin$_{R126W}$-GrpEL1$_{WT}$ and GrpEL1 predicted structures.

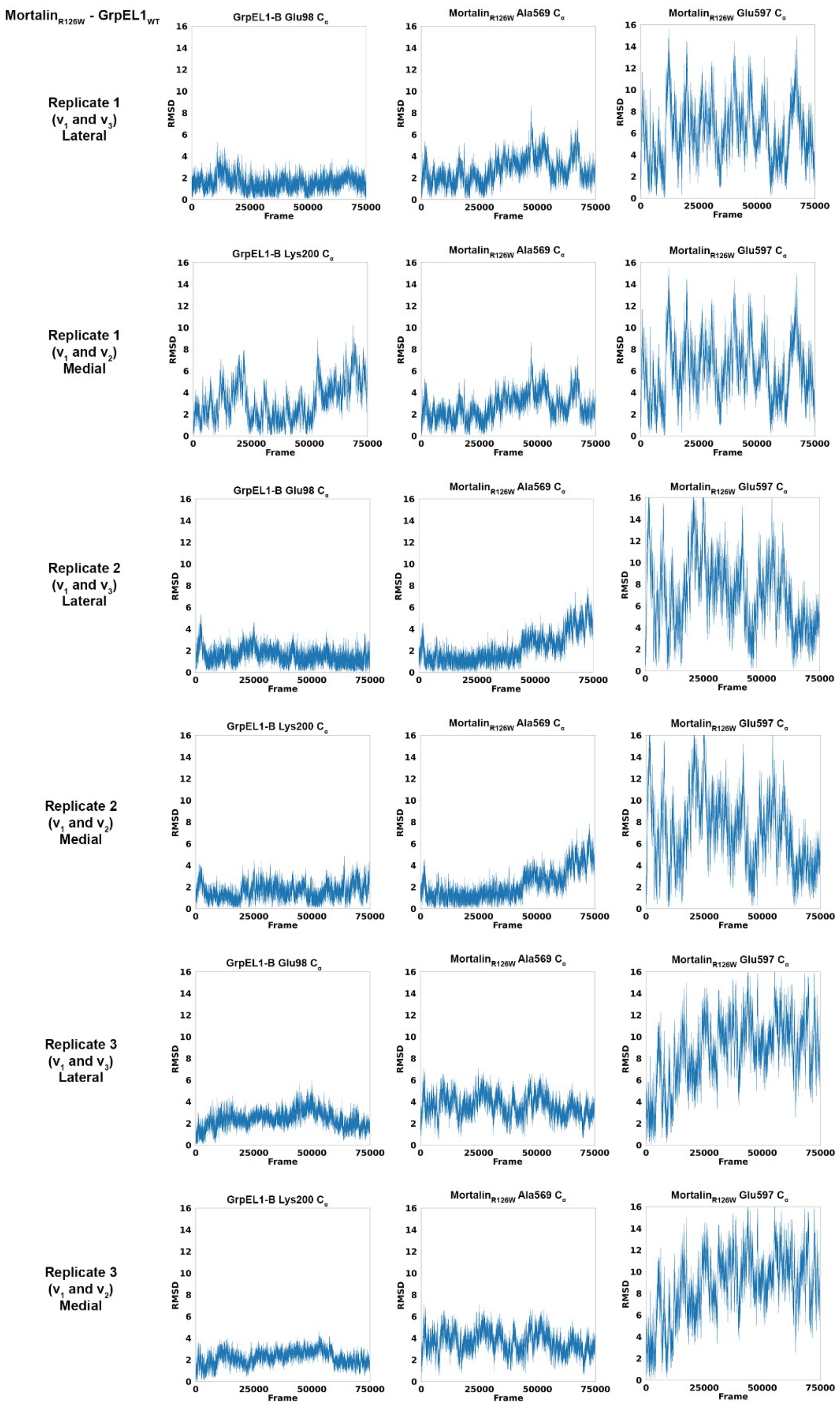

Supplementary Figure 22. RMSD analysis of vectors v_1, v_2 and v_3 in mortalin$_{R126W}$-GrpEL1$_{WT}$.[55,58] RMSD analyses were carried out on Cα's, defined in **Supplementary Figure 14**, used in the assessment of lateral and medial motions across the three mortalin$_{R126W}$-GrpEL1$_{WT}$ all-atom simulations.

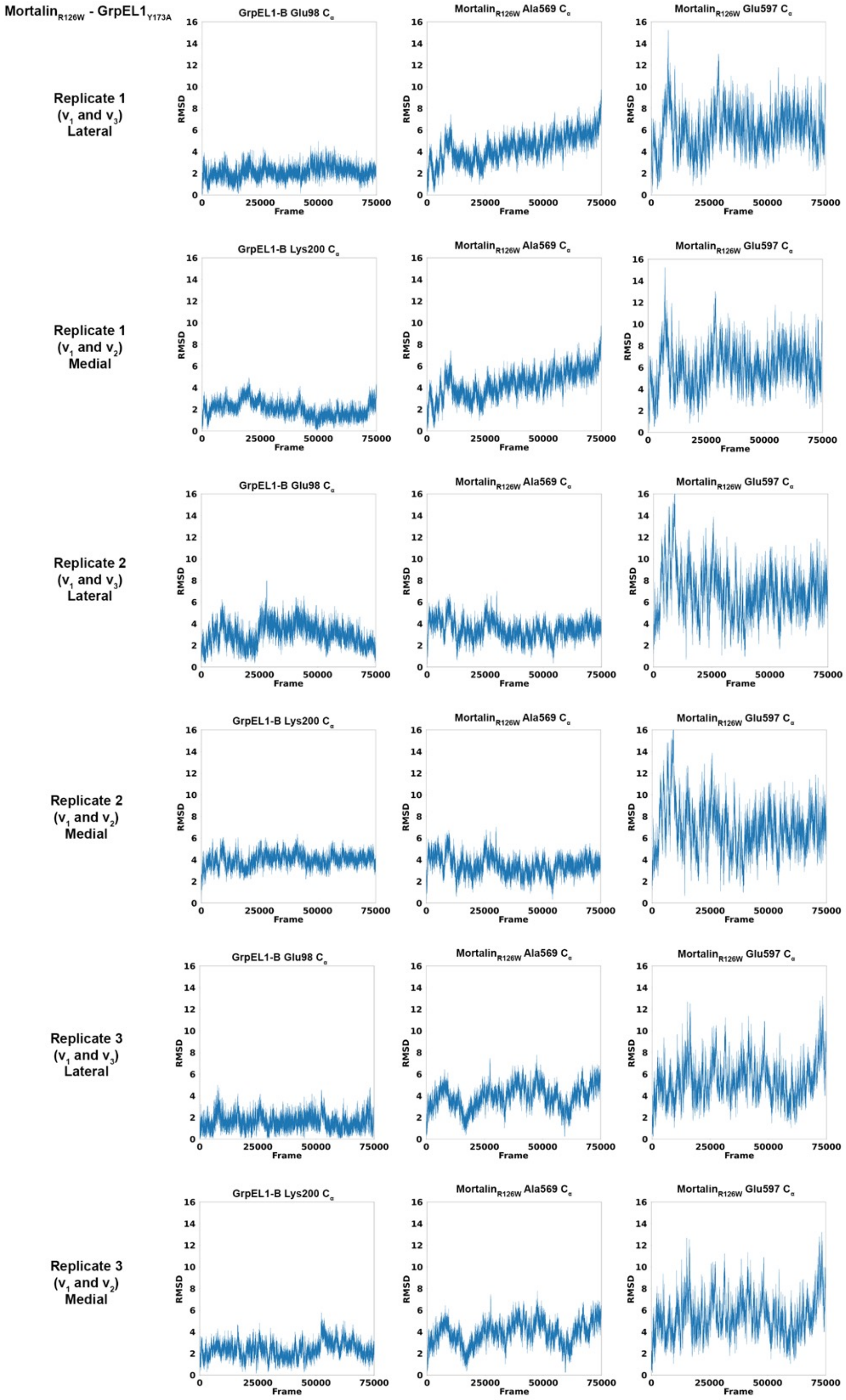

Supplementary Figure 23. RMSD analysis of vectors v_1, v_2, and v_3 in mortalin$_{R126W}$-GrpEL1$_{Y173A}$.[55,58] RMSD analyses were carried out on Cα's, defined in **Supplementary Figure 14**, used in the assessment of lateral and medial motions across the three mortalin$_{R126W}$-GrpEL1$_{Y173A}$ all-atom simulations.

	Mortalin$_{R126W}$-GrpEL1$_{WT}$	Mortalin$_{R126W}$-GrpEL1$_{Y173A}$	
**Data Collection**			
Magnification	130kx	165kx	
Voltage (kV)	300	300	
Spherical Aberration (mm)	2.7	2.7	
Electron Exposure ($e^-/Å^2$)	60	60	
Defocus range (μm)	-1.0 to -2.5	-1.0 to -2.5	
Pixel size (Å, Physical/Digital)	0.935	0.735	
Energy Filter Slit Width (eV)	10	10	
Movies	4570	3669	
**Map Statistics and Post-Processing**		**Mortalin$_{R126W}$-GrpEL1$_{Y173A}$**	**Mortalin$_{R126W}$-GrpEL1$_{Y173A}$-lid**
Accession Codes (EMDB, PDB)	EMD-44675, 9BLS	EMD-44676, 9BLT	EMD-44677, 9BLU
Symmetry imposed	C1	C1	C1
Map Resolution (Å)	2.96	3.38	3.38
Local resolution range for 75% of voxels	5.825	7.874	7.326
Local resolution range (model)	2.579 - 31.802	3.014 - 52.055	2.999 - 47.490
Map sharpening B factor ($Å^2$)	97.9	83.2	85.7
Map sharpening method	DeepEMhancer	DeepEMhancer	DeepEMhancer
Table Name:	table1	table1	table1
**Model Statistics and Validation**			
Model composition			
Non-hydrogen atoms	7113	7099	6378
Protein	921	921	831
Nucleic acids	0	0	0
Ligands	0	0	0
Waters	0	0	0
R.M.S deviations			
Length (Å)	0.003	0.003	0.003
Angles (°)	0.503	0.505	0.452
MolProbity score	2.41	2.11	1.92
MolProbity Clashscore	8.55	8.29	6.89
CaBLAM (% outliers)	2.32	1.66	2.21
Rotamer outliers (%)	5.30	2.72	3.18
Cis peptides (#, %)	0.0/0.0	0.0/0.0	0.0/0.0
Ramachandran Plot			
Favored	94.09	95.18	97.08
Allowed	5.91	4.82	2.92
Outliers	0.0	0.0	0.0

Table 1. CryoEM data collection and refinement statistics of mortalin$_{R126W}$-GrpEL1$_{WT}$, mortalin$_{R126W}$-GrpEL1$_{Y173A}$, and mortalin$_{R126W}$-GrpEL1$_{Y173A}$-lid.
