# Supplementary material for: Structural insights into GrpEL1-mediated nucleotide and substrate release of human mitochondrial Hsp70
Source: Nat Commun. 2024 Dec 30;15:10815. doi: 10.1038/s41467-024-54499-1 (PMC11685456; doi:10.1038/s41467-024-54499-1)
Supplement: Supplementary file 2 — Description of Additional Supplementary Files [file 41467_2024_54499_MOESM2_ESM.pdf]

Description of Additional Supplementary Files

File Name: Supplementary Movie 1

Description: Anisotropic network modelling (ANM) analysis of mortalin$_{R126W}$-GrpEL1$_{WT}$. Ten ANM modes of the mortalin$_{R126W}$-GrpEL1$_{WT}$ structure were visualized using VMD[58] and highlight the flexibility of the mortalin$_{R126W}$ SBD. Areas of high motion are colored in red, and areas of low motion are colored in blue.

File Name: Supplementary Movie 2

Description: Anisotropic network modelling (ANM) analysis of mortalin$_{R126W}$-GrpEL1$_{Y173A}$. Ten ANM modes of the mortalin$_{R126W}$-GrpEL1$_{Y173A}$ structure were visualized using VMD[58] and highlight the flexibility of the mortalin$_{R126W}$ SBD. Areas of high motion are colored in red, and areas of low motion are colored in blue.

File Name: Supplementary Movie 3

Description: Replicate 1 of all-atom molecular dynamics simulation of mortalin$_{R126W}$-GrpEL1$_{WT}$. The mortalin$_{R126W}$-GrpEL1$_{WT}$ all-atom molecular dynamics simulation was performed[55-57] as described in the *Methods* section. The mortalin NBD is colored in yellow, the interdomain linker in grey, the SBD in orange, substrate in cyan, GrpEL1-A in dark blue, and GrpEL1-B in purple. The alpha carbons of residues 569 and 597 in mortalin (vector v_1), alpha carbons of residue 569 in mortalin and residue 200 in GrpEL1-B (vector v_2), and alpha carbons of residue 569 in mortalin and residue 98 in GrpEL1-B (vector v_3), are represented as green spheres.

File Name: Supplementary Movie 4

Description: Replicate 2 of all-atom molecular dynamics simulation of mortalin$_{R126W}$-GrpEL1$_{WT}$. The mortalin$_{R126W}$-GrpEL1$_{WT}$ all-atom molecular dynamics simulation was performed[55-57] as described in the *Methods* section. The mortalin NBD is colored in yellow, the interdomain linker in grey, the SBD in orange, substrate in cyan, GrpEL1-A in dark blue, and GrpEL1-B in purple. The alpha carbons of residues 569 and 597 in mortalin (vector v_1), alpha carbons of residue 569 in mortalin and residue 200 in GrpEL1-B (vector v_2), and alpha carbons of residue 569 in mortalin and residue 98 in GrpEL1-B (vector v_3), are represented as green spheres.

File Name: Supplementary Movie 5

Description: Replicate 3 of all-atom molecular dynamics simulation of mortalin$_{R126W}$-GrpEL1$_{WT}$. The mortalin$_{R126W}$-GrpEL1$_{WT}$ all-atom molecular dynamics simulation was performed[55-57] as described in the *Methods* section. The mortalin NBD is colored in yellow, the

interdomain linker in grey, the SBD in orange, substrate in cyan, GrpEL1-A in dark blue, and GrpEL1-B in purple. The alpha carbons of residues 569 and 597 in mortalin (vector v_1), alpha carbons of residue 569 in mortalin and residue 200 in GrpEL1-B (vector v_2), and alpha carbons of residue 569 in mortalin and residue 98 in GrpEL1-B (vector v_3), are represented as green spheres.

File Name: Supplementary Movie 6

Description: Replicate 1 of all-atom molecular dynamics simulation of mortalin$_{\mathbf{R126W}}$-GrpEL1$_{\mathbf{Y173A}}$. The mortalin$_{R126W}$-GrpEL1$_{Y173A}$ all-atom molecular dynamics simulation was performed[55-57] as described in the *Methods* section. The mortalin NBD is colored in yellow, the interdomain linker in grey, the SBD in orange, substrate in cyan, GrpEL1-A in dark blue, and GrpEL1-B in purple. The alpha carbons of residues 569 and 597 in mortalin (vector v_1), alpha carbons of residue 569 in mortalin and residue 200 in GrpEL1-B (vector v_2), and alpha carbons of residue 569 in mortalin and residue 98 in GrpEL1-B (vector v_3), are represented as green spheres.

File Name: Supplementary Movie 7

Description: Replicate 2 of all-atom molecular dynamics simulation of mortalin$_{\mathbf{R126W}}$-GrpEL1$_{\mathbf{Y173A}}$. The mortalin$_{R126W}$-GrpEL1$_{Y173A}$ all-atom molecular dynamics simulation was performed[55-57] as described in the *Methods* section. The mortalin NBD is colored in yellow, the interdomain linker in grey, the SBD in orange, substrate in cyan, GrpEL1-A in dark blue, and GrpEL1-B in purple. The alpha carbons of residues 569 and 597 in mortalin (vector v_1), alpha carbons of residue 569 in mortalin and residue 200 in GrpEL1-B (vector v_2), and alpha carbons of residue 569 in mortalin and residue 98 in GrpEL1-B (vector v_3), are represented as green spheres.

File Name: Supplementary Movie 8

Description: Replicate 3 of all-atom molecular dynamics simulation of mortalin$_{\mathbf{R126W}}$-GrpEL1$_{\mathbf{Y173A}}$. The mortalin$_{R126W}$-GrpEL1$_{Y173A}$ all-atom molecular dynamics simulation was performed[55-57] as described in the *Methods* section. The mortalin NBD is colored in yellow, the interdomain linker in grey, the SBD in orange, substrate in cyan, GrpEL1-A in dark blue, and GrpEL1-B in purple. The alpha carbons of residues 569 and 597 in mortalin (vector v_1), alpha carbons of residue 569 in mortalin and residue 200 in GrpEL1-B (vector v_2), and alpha carbons of residue 569 in mortalin and residue 98 in GrpEL1-B (vector v_3), are represented as green spheres.
