# Supplementary material for: Structural insights into GrpEL1-mediated nucleotide and substrate release of human mitochondrial Hsp70
Source: Nat Commun. 2024 Dec 30;15:10815. doi: 10.1038/s41467-024-54499-1 (PMC11685456; doi:10.1038/s41467-024-54499-1)
Supplement: Supplementary file 11 — Reporting Summary [file 41467_2024_54499_MOESM11_ESM.pdf]

nature portfolio

Corresponding author(s): Mark Herzik Jr.

Last updated by author(s): Oct 7, 2024

Reporting Summary

Nature Portfolio wishes to improve the reproducibility of the work that we publish. This form provides structure for consistency and transparency in reporting. For further information on Nature Portfolio policies, see our Editorial Policies and the Editorial Policy Checklist.

Statistics

For all statistical analyses, confirm that the following items are present in the figure legend, table legend, main text, or Methods section.

n/a	Confirmed	
☒	☐	The exact sample size (*n*) for each experimental group/condition, given as a discrete number and unit of measurement
☒	☐	A statement on whether measurements were taken from distinct samples or whether the same sample was measured repeatedly
☒	☐	The statistical test(s) used AND whether they are one- or two-sided<br>*Only common tests should be described solely by name; describe more complex techniques in the Methods section.*
☒	☐	A description of all covariates tested
☒	☐	A description of any assumptions or corrections, such as tests of normality and adjustment for multiple comparisons
☐	☒	A full description of the statistical parameters including central tendency (e.g. means) or other basic estimates (e.g. regression coefficient) AND variation (e.g. standard deviation) or associated estimates of uncertainty (e.g. confidence intervals)
☒	☐	For null hypothesis testing, the test statistic (e.g. *F*, *t*, *r*) with confidence intervals, effect sizes, degrees of freedom and *P* value noted<br>*Give P values as exact values whenever suitable.*
☒	☐	For Bayesian analysis, information on the choice of priors and Markov chain Monte Carlo settings
☒	☐	For hierarchical and complex designs, identification of the appropriate level for tests and full reporting of outcomes
☒	☐	Estimates of effect sizes (e.g. Cohen's *d*, Pearson's *r*), indicating how they were calculated

Our web collection on statistics for biologists contains articles on many of the points above.

Software and code

Policy information about availability of computer code

Data collection	CryoEM Data Collection - Titan Krios G4 operating at 300 kV equipped with a Selectris-X energy filter using EPU2 (ThermoFisher Scientific).<br>Size Exclusion Chromatograms - A Superdex 200 10/300 Increase column (Cytiva) connected to an AKTA pure protein purification system (Cytiva) running UNICORN (version 7.6) was used to collect size exclusion chromatography data.<br>SEC-MALS Data - A Superdex 200 10/300 Increase (Cytiva) connected to a 1260 Infinity II HPLC (Agilent) running ASTRA (version 8.2.1) was used to collect SEC-MALS data.<br>Anisotropic Network Modeling (ANM) - ANM calculations were parsed using ProDy (version 2.0)<br>All-atom Molecular Dynamics Simulations - All-atom molecular dynamics simulations were performed using the CUDA memory-optimized version of NAMD (version 2.14) and CHARMM36m force fields.
Data analysis	CryoEM Data Analysis - cryoSPARC (version 4.3.1)<br>MALS Data Analysis - ASTRA (version 8.2.1)<br>Protein Structure Prediction - AlphaFold2 (version 2.3.2)<br>Model Building and Editing - COOT (version 0.9.8.8)<br>Model Refinement - PHENIX (version 1.21)<br>Model and Density Visualization - UCSF ChimeraX (version 1.7.0)<br>Multiple Sequence Alignments - Clustal Omega (version 1.2.4)<br>All-atom Molecular Dynamics Simulations - NAMD Multicore Cuda (version 2.14)<br>Molecular Dynamics Simulations Analysis - Visual Molecular Dynamics (VMD) (version 1.9.4.a57)<br>Molecular Dynamics Simulations Analysis - MATLAB (version 2023b)<br>Code Generation and Analysis - Python (version 3.11)

Anisotropic Network Modeling Analysis - Visual Molecular Dynamics (VM) (version 1.9.4.a57) with the Normal Mode Wizard (NMWiz) plugin (version 1.9.1)

For manuscripts utilizing custom algorithms or software that are central to the research but not yet described in published literature, software must be made available to editors and reviewers. We strongly encourage code deposition in a community repository (e.g. GitHub). See the Nature Portfolio guidelines for submitting code & software for further information.

Data

Policy information about availability of data

All manuscripts must include a data availability statement. This statement should provide the following information, where applicable:

- Accession codes, unique identifiers, or web links for publicly available datasets
- A description of any restrictions on data availability
- For clinical datasets or third party data, please ensure that the statement adheres to our policy

The data supporting this study are available from the corresponding author upon request. All models and associated cryoEM maps have been deposited into the Electron Microscopy Data Bank (EMDB) and the PDB. The depositions include final maps, unsharpened maps, half maps, and associated FSC curves. The cryoEM maps have been deposited in the Electron Microscopy Data Bank (EMDB) under accession codes EMD-44675 [https://www.ebi.ac.uk/pdbe/entry/emdb/EMD-44675] (MortalinR126W-GrpEL1WT); EMD-44676 [https://www.ebi.ac.uk/pdbe/entry/emdb/EMD-44676] (MortalinR126W-GrpEL1Y173A); and EMD-44677 [https://www.ebi.ac.uk/pdbe/entry/emdb/EMD-44677] (MortalinR126W-GrpEL1Y173A-lid). The atomic coordinates have been deposited in the Protein Data Bank (PDB) under accession codes PDB: 9BLS [https://doi.org/10.2210/pdb9BLS/pdb] (MortalinR126W-GrpEL1WT); PDB: 9BLT [https://doi.org/10.2210/pdb9BLT/pdb] (MortalinR126W-GrpEL1Y173A); and PDB: 9BLU [https://doi.org/10.2210/pdb9BLU/pdb] (MortalinR126W-GrpEL1Y173A-lid). Source data are provided with this paper.

The atomic coordinates of referenced structures are deposited in the PDB under the following codes: 5OBW [http://doi.org/10.2210/pdb5OBW/pdb]; 8BG3 [http://doi.org/10.2210/pdb8BG3/pdb]; 4KBO [http://doi.org/10.2210/pdb4KBO/pdb]; 6NHK [http://doi.org/10.2210/pdb6NHK/pdb]; 4ANI [http://doi.org/10.2210/pdb4ANI/pdb]; 4EZW [http://doi.org/10.2210/pdb4EZW/pdb]; 2GUZ [http://doi.org/10.2210/pdb2GUZ/pdb]; 1DKG [http://doi.org/10.2210/pdb1DKG/pdb]; 4PO2 [http://doi.org/10.2210/pdb4PO2/pdb]; 3N8E [http://doi.org/10.2210/pdb3N8E/pdb]; 6ZHI [http://doi.org/10.2210/pdb6ZHI/pdb]; 4JNF [http://doi.org/10.2210/pdb4JNF/pdb]; 4R5L [http://doi.org/10.2210/pdb4R5L/pdb]; 4F01[http://doi.org/10.2210/pdb4F01/pdb].

Research involving human participants, their data, or biological material

Policy information about studies with human participants or human data. See also policy information about sex, gender (identity/presentation), and sexual orientation and race, ethnicity and racism.

Reporting on sex and gender	N/A
Reporting on race, ethnicity, or other socially relevant groupings	N/A
Population characteristics	N/A
Recruitment	N/A
Ethics oversight	N/A

Note that full information on the approval of the study protocol must also be provided in the manuscript.

Field-specific reporting

Please select the one below that is the best fit for your research. If you are not sure, read the appropriate sections before making your selection.

☒ Life sciences ☐ Behavioural & social sciences ☐ Ecological, evolutionary & environmental sciences

For a reference copy of the document with all sections, see nature.com/documents/nr-reporting-summary-flat.pdf

Life sciences study design

All studies must disclose on these points even when the disclosure is negative.

Sample size	Not applicable; no experiments including statistical sampling were performed in this study
Data exclusions	No data was excluded from our study

Replication	All molecular dynamics simulations were ran in triplicate to evaluate reproducibility. Variability across replicates is described by RMSD analysis of individual domains (Supplementary Figures 18 and 19) and RMSD analysis of vectors v1-v3 (Supplementary Figures 22 and 23).
Randomization	Randomization was not relevant to our study
Blinding	Blinding was not relevant to our study

Reporting for specific materials, systems and methods

We require information from authors about some types of materials, experimental systems and methods used in many studies. Here, indicate whether each material, system or method listed is relevant to your study. If you are not sure if a list item applies to your research, read the appropriate section before selecting a response.

Materials & experimental systems

n/a	Involved in the study
☒	☐ Antibodies
☒	☐ Eukaryotic cell lines
☒	☐ Palaeontology and archaeology
☒	☐ Animals and other organisms
☒	☐ Clinical data
☒	☐ Dual use research of concern
☒	☐ Plants

Methods

n/a	Involved in the study
☒	☐ ChIP-seq
☒	☐ Flow cytometry
☒	☐ MRI-based neuroimaging

Plants

Seed stocks	*Report on the source of all seed stocks or other plant material used. If applicable, state the seed stock centre and catalogue number. If plant specimens were collected from the field, describe the collection location, date and sampling procedures.*
Novel plant genotypes	*Describe the methods by which all novel plant genotypes were produced. This includes those generated by transgenic approaches, gene editing, chemical/radiation-based mutagenesis and hybridization. For transgenic lines, describe the transformation method, the number of independent lines analyzed and the generation upon which experiments were performed. For gene-edited lines, describe the editor used, the endogenous sequence targeted for editing, the targeting guide RNA sequence (if applicable) and how the editor was applied.*
Authentication	*Describe any authentication procedures for each seed stock used or novel genotype generated. Describe any experiments used to assess the effect of a mutation and, where applicable, how potential secondary effects (e.g. second site T-DNA insertions, mosiacism, off-target gene editing) were examined.*
